# Colostrum extracellular vesicles are neuroprotective in models of Parkinson's disease

**DOI:** 10.7150/thno.128257

**Published:** 2026-01-22

**Authors:** Dawson Hollingsworth, Shefali Srivastava, Samia Akter, Mohit Kumar, Soumya Sagar Dey, Sudipta Panja, Xiaoqing Du, Arnab Saha, Pravin Yeapuri, Shaurav Bhattarai, Emma G. Foster, Rana Kadry, Nada Fayez, Davina Oludipe, Emma Ehrenkranz, R Lee Mosley, John Oehlerking, Keith Swarts, Guoku Hu, Howard E. Gendelman, Susmita Sil

**Affiliations:** 1 Department of Pharmacology and Experimental Neuroscience, University of Nebraska Medical Center, Omaha, Nebraska, USA.; 2 Oehlerking Farm, Omaha, Nebraska, USA.

**Keywords:** Parkinson's disease, tyrosine hydroxylase neurons, regulatory T cells, neuroprotection, anti-inflammatory activities

## Abstract

**Rationale**: Parkinson's disease (PD) is a progressive neurodegenerative disorder that affects movement, muscle control, and balance. Effective therapeutic options for this condition are limited. Natural therapies, including lifestyle changes, probiotics, and muscle relaxants, have received attention for symptomatic relief. Colostrum, particularly its extracellular vesicles (C-EVs), has emerged as a promising nutraceutical with the potential to improve therapeutic outcomes in divergent diseases.

**Methods:** We purified and characterized (C-EVs) as a putative cell-based therapy. Theranostic (biodistribution, diagnostic, and therapeutic) efficacy studies were performed in C-EV-treated mice intoxicated with methyl-4-phenyl-1,2,3,6-tetrahydropyridine (MPTP). The C-EV tissue distribution, anti-inflammatory and neurorestorative activities were examined. These include transcriptomic, immune, and neuroprotective profiles linked to disease outcomes.

**Results**: C-EV-treated MPTP mice showed reduced microglial activation and restored neuronal responses. RNA sequencing and transcriptomic analyses have demonstrated reduced immune cell recruitment and activation in the disease-affected brain subregions. The activation of canonical inflammasomes, pro-inflammatory cytokines, and chemokine expression was controlled. C-EV treatment reduced the levels of disease-associated immune-regulatory transcription factors. Simultaneously, Treg-associated adaptive immune responses increased. Multiple C-EV-miR-isolated immune regulatory cargos are linked to neuroinflammation and nigral preservation. C-EVs loaded with miR-20a-5p, miR-23b-3p, let-7a-5p, miR-22-3p, and miR-30a-3p mimics attenuated pro-inflammatory cytokines in activated microglia.

**Conclusions**: C-EVs elicit neuroprotective responses in MPTP-intoxicated mice. These responses control microglial activation and facilitate neuroprotective responses.

## Introduction

Innate immune, mitochondrial, and glial dysfunction are pathological features of Parkinson's disease (PD). Each of these factors affects dopaminergic metabolism, iron content, and oxidative stress 1. Dysfunction of adaptive immune responses is linked to an increase in HLA-DR+ and CD45RO^+^ memory cells, reductions in CD4^+^ regulatory T cells (Treg), and decreases in B cells, which signal generalized inflammatory responses 2-4. The cumulative effect is the dysfunction and consequent loss of the nigral dopaminergic neurons. PD-related immune abnormalities are expected because genetic factors associated with PD risk are related to immune dysregulation 5-9. Preclinical studies have shown a connection between inflammation control and the survival of dopaminergic neurons 10-12. These include immune-regulating agents such as granulocyte-macrophage colony-stimulating factor (GM-CSF) and interleukin-2 (IL-2). Both induce anti-inflammatory Tregs that manage neuroinflammation and protect neurons 10-12. Recently, extracellular vesicles (EVs), comprising microvesicles, exosomes, and apoptotic bodies secreted by a spectrum of cell types, have demonstrated protective and pathological activities. The former responses improve intercellular communication 13, tissue regeneration, and therapeutic delivery 14-17. Stem cell-derived EVs, for example, can ameliorate pulmonary hypertension, kidney, hepatic, musculoskeletal, cardiovascular, and neurological injuries and cancer 17. EVs facilitate improved disease outcomes by delivering disease-combating miRNAs and proteins 16, 18-23. Clinical trials of EVs for melanoma 25, non-small lung (NCT01159288), colon 26, pancreatic, and other cancers (NCT03608631), as well as diabetes (NCT02138331), stroke (NCT03384433), and lung diseases (NCT03857841), have begun to harness their potential therapeutic role 17, 24, 25. EVs can also drive disease pathology by transferring proteins, RNAs to distant cells, promoting inflammation, facilitating cancer metastasis, and spreading toxic proteins like amyloid. Thus, EVs are "Janus-faced" entities as they can be engineered or selected to ameliorate disease based on their cell and tissues of origins, and source of recovered fluids largely acting as therapeutic, anti-inflammatory, and regenerative agents or in driving disease based on their formation, timing, and disease presence 91-21.

Colostrum EVs (C-EVs) have recently garnered interest owing to their disease-combating properties 26, 27. Their use has been buoyed by their high safety profiles, failure to elicit host immune responses, significant recovery, and immune regulatory responses 28-30. C-EVs are rich in bioactive proteins, lipids, RNAs, and metabolites that elicit potent anti-inflammatory effects 31-34. As the first milk produced postpartum, colostrum is enriched with immunoglobulins, antimicrobial peptides, cytokines, and growth factors that provide immune protection and support the development of the neonatal immune system 35, 36. In addition, colostrum contains a high abundance of EVs and bioactive miRNAs 31, 37. Rich in bioactive proteins, they can control inflammatory activities, reduce apoptosis, promote epithelial cell proliferation, and overcome barrier dysfunctions 32, 34. Notably, C-EVs regulate human macrophage activation by increasing interleukin-10 (IL-10) secretion and decreasing the expression of necrosis factor-alpha (TNF-α) and IL-1β levels 38. C-EVs can also promote wound healing 27, including the repair of epithelial cells 27 and transport to the brain 39. They are also involved in signal transduction, cell cycle progression, and immune responses 40-42. Considering their broad favorable tissue and cell restorative activities coupled with their broad biodistribution, scalability, and disease-combating properties, we reasoned that they may be harnessed as neuroprotectants 43. This idea supports its use, further supported by its ability to ameliorate degenerative diseases 44.

Thus, we investigated the potential utility of C-EVs in PD using rodent models of human disease. C-EVs were administered to methyl-4-phenyl-1,2,3,6-tetrahydropyridine (MPTP) intoxicated mice model of PD. In these experiments, mice were administered EVs before, on the day of, and after MPTP administration and were then sacrificed. Brains were evaluated two and seven days after MPTP administration. As an inflammatory response precedes neurodegeneration in mice, both conditions were assessed. Anti-inflammatory responses were demonstrated by controlling microglial activation. Transcriptomic tests have demonstrated reduced immunocyte recruitment and pro-inflammatory responses. All these findings were consistent with decreased inflammasome activation. Each was associated with reduced levels of regulatory transcription factors (TF). The links between the control of neuroinflammation and neuroprotection were confirmed by the protection of tyrosine hydroxylase (TH+) nigral dopaminergic neurons. Up to 33% TH+ neuronal preservation was recorded in C-EV-treated MPTP mice. These results were associated with reduced neuronal cell death and neuroinflammation. The enrichment of anti-inflammatory and neuroprotective miRNAs affirms the therapeutic potential of C-EV.

## Materials and Methods

### Theranostic studies

C57BL/6 (6-8 weeks old, male) were purchased from Jackson Laboratories (Bar Harbor, ME, USA) and cared for according to the animal care guidelines issued by the National Institutes of Health and approved by the Institutional Animal Care and Use Committee of the University of Nebraska Medical Center. A pilot study was conducted to assess the biodistribution of EVs using positron emission tomography (PET) and an in vivo imaging system (IVIS). After confirming C-EV brain targeting, separate groups of mice were assessed to determine the C-EV dose required for neuroprotection. The mice were intravenously injected with 10^11^ EVs. EV doses were administered one and three days before and on the day of MPTP intoxication. This was followed by the first sacrifice two days after intoxication to assess neuroinflammation, which included studies of brain mononuclear phagocytes (MP; brain-derived macrophages and microglia) function using transcriptomic analysis. Additional evaluations of regulatory T cell (Treg) numbers were conducted by flow cytometry. On day seven following MPTP intoxication, neuronal counts were determined. An extension study was then performed. This was achieved by the continuous administration of C-EVs. Mice were intravenously injected with 10^11^ C-EVs starting three days before and ending four days after MPTP. The regimen consisted of eight consecutive doses administered over eight days. The mice were administered 100 μL phosphate-buffered saline (PBS) without C-EVs as a control. In all animal experiments, MPTP-intoxicated mice received four subcutaneous doses of MPTP. MPTP hydrochloride (MPTP-HCl) was administered in PBS (16 mg/kg of MPTP-free base) over eight hours. On day two, MP immune responses were collected. One hemisphere was used to investigate transcriptomic changes, whereas the other, which included the midbrain and striatum, was used for regional immunohistochemical analyses. Neuronal survival was evaluated on day seven. The midbrain and striatum were assessed for TH-expressing somas and terminals, respectively. Whole blood, brain, and spleen samples were collected to assess the Treg responses. All experiments were performed in a blinded manner.

### EV Isolation

Extracellular vesicles (EVs) were isolated using differential centrifugation 45. Briefly, 20 mL bovine colostrum (day 1) was obtained from the Oehlerking Farm, Omaha, Nebraska. Colostrum was diluted 1:1.5 by volume with PBS and centrifuged at 5000 × g for 30 min. Ethylenediaminetetraacetic acid (EDTA) was added to the supernatant at a 1:1 ratio to yield 100 mM EDTA, and the mixture was placed on ice for 15 min. This was followed by centrifugation at 12,000× g for 1 h to remove chelated calcium and casein. The supernatant was centrifuged at 70,000g for two hours to remove large debris and protein contaminants, followed by filtration through a 0.22 µm filter. Next, the supernatant EVs were pelleted at 100,000 × g for 1 h, washed in PBS, and re-pelleted at 100,000 × g for 1 h. The pellet was resuspended in 600 μL of PBS and characterized. All centrifugation steps above 300 g were performed at 4°C in an SW 32 Ti, Beckman Coulter swing bucket rotor in a Discovery 90SE ultracentrifuge (Beckman, IN, USA) with acceleration set to “max” and deceleration set to “3.” Additionally, C-EVs were isolated using the OptiPrep method, with 50 mL of defatted aliquoted colostrum. Each 12 mL aliquot was centrifuged at 4700 g for 20 min at 4 °C, which removed fat debris. Next, the colostrum was centrifuged at 2000 g for 20 min at 4 °C, and the supernatant was filtered through Whatman grade 1 filter paper. An equal volume of 0.25 M EDTA, pH 7, was added to the filtrate, which was then incubated on ice for 20 min. The pH was adjusted to 4.6 using 6 N acetic acid. The filtrate was then ultracentrifuged at 65,000 × g for 1.5 h, followed by filtration using 0.45 and 0.22-micron filters. After filtration, gradient ultracentrifugation was performed using 40, 30, 20, 10, and 5% Opti prep dilutions at 186,000 × g for 18 h at 10 °C. Experiments were performed in an SW 41 Ti swinging bucket- rotor using a Discovery 90SE ultracentrifuge (Beckman Coulter, IN, USA; acceleration “max,” deceleration “3”). Each fraction was collected and washed at 110,000 × g for 90 min at 4 °C. EVs were prepared at concentrations of 10¹¹-10¹² particles/mL. The resulting pellets were resuspended in 1 mL of PBS, and C-EV purity was validated 16, 19, 46, 47. The recovered EV pellet was resuspended in 1 mL PBS and fully characterized.

### C-EV characterization

C-EVs were diluted 1:1000 in PBS before characterization. Dynamic light scattering (DLS) was performed in triplicate for each sample using a Zetasizer Nano (Malvern Panalytical Ltd.). The settings were as follows: Dispersant; PBS; temperature, 25 °C; viscosity, one cP; Material Detected, polystyrene latex RI, 1.590; absorption, 0.010. Nanoparticle tracking analysis 19, 48 was performed using an NS300 Nanosight (Malvern Panalytical Ltd.) with a green 532 nm laser. NTA 3.4 Build 3.4.4 software was used to acquire and analyze all measurements. The system was set to the following specifications: frame rate, 24,9825 fps; Slider Gain, 219; Shutter Speed, 30.8 ms; Screen Gain, 1.8; Camera Level, 14; and Detection Limit, 5. The chamber was flushed with 1 mL of PBS before EV analysis and washed with 1 mL of PBS between the samples. Samples (500 µL) were passed through the chamber before measurement, and the experiment was performed in quadruplicate. The number of C-EVs was determined and expressed as EVs/mL.

### Transmission and cryogenic electron microscopy (TEM and cryo-EM)

TEM was performed as previously described 19. The isolated EVs were resuspended in glutaraldehyde and added to 200-mesh Formvar copper grids for 5 min at room temperature. After incubation, the grids were transferred to a uranyl acetate solution for negative staining. The stained EVs were washed with PBS and dried at room temperature prior to imaging. Images were acquired using a Hitachi H-7500 electron microscope (Hitachi, Tokyo, Japan) at 200 kV magnification. Cryo-EM imaging was performed at the Hormel Institute at the University of Minnesota Medical Research Center contract laboratories.

### Biodistribution

#### Positron Emission Tomography (PET)

C-EV biodistribution was characterized by PET and computed tomography (CT) in BALB/c male (six to eight weeks old) mice using ^64^Cu-labeled EV. Briefly, a 500 mM solution of copper (^64^Cu) chloride and 0.25 µM of EDTA (pH 7) was prepared using Milli-Q water. The copper chloride solution was added dropwise to the EDTA solution, and the mixture was incubated at room temperature with constant shaking to prepare the EV-Cu-EDTA complex. The solution was centrifuged at 10,000 × g for 30 min to remove the unconjugated large particles, and the supernatant was collected carefully without disturbing the pellet. A 10 kDa micron filter was used to remove unbound copper and concentrate the labeled EVs, which were resuspended in 100 µL of PBS. A dose of 1000 µCi/kg body weight was intravenously injected. The mice were anesthetized with isoflurane, and images were obtained 6 h post-injection using PET imaging (MOLECUBE β-CUBE; Ghent, Belgium). The following parameters were used for PET imaging: 64 projections at 15 s per projection over a 360° rotation with a 48 mm radius (field of view = 59 mm^2^). VivoQuant 3.5 software (Invicro, Boston, MA, USA) was used for the co-registration of the 3D CT. The mice were sacrificed immediately after the imaging. The organs were harvested, weighed, and their radioactivity was measured using gamma scintillation spectrometry (Hidex Automatic Gamma Counter, Turku, Finland). The following equation was used to determine the radioactivity count percentage: Radioactivity count (%/ g tissue) = (organ radioactivity count × 100)/ (total radioactivity count × organ weight).

#### *In Vivo* Imaging System (IVIS)

Six-to eight-week-old- male C57BL/6J mice were administered a single intravenous injection via the tail vein of 100 µl of 10¹¹/mL EVs. Twenty-four- hours post-injection-, the animals were deeply anesthetized and euthanized according to institutional guidelines, and the brain, heart, lungs, liver, spleen, kidneys, and gut were dissected, rinsed in cold PBS, and gently blotted dry. The organs were placed in a predefined orientation in a black, non-reflective- imaging tray and imaged (PerkinElmer, Waltham, MA, USA) with excitation spanning 535-570 nm and emission filters spanning 580-620 nm. Images were acquired using identical exposure time, binning, f-stop-, and field of view settings. Fluorescence signals were quantified in Living Image 4.5 software from images acquired by the Living Image 4.7.4 system 16, 49

#### Western blotting

C-EVs were incubated with Triton TE buffer at 37 °C for 10 minutes. Protein concentration was measured using the Pierce BCA Protein Assay Kit (Cat. No. 23235, ThermoFisher Scientific, MA, USA). Fresh EVs were diluted to a uniform concentration and mixed with 2X Laemmli buffer (Cat. No. 161 0737, Bio-Rad, CA, USA) and 7.5% 2-mercaptoethanol to denature the proteins. The samples were then incubated at 99 °C for 5 min. Brain tissues from different groups were lysed in radioimmunoprecipitation assay (RIPA) lysis buffer and sonicated. The lysates were centrifuged, and the supernatants were collected for further analysis. Protein concentration was measured using a Micro BCA Protein Assay Kit, and the samples were subjected to electrophoresis and western blotting 29, 30. Briefly, equal amounts of protein samples were loaded and separated by electrophoresis on 10-15% polyacrylamide gels under reducing conditions. After electrophoresis, the proteins were transferred to polyvinylidene fluoride (PVDF) membranes (Cat. No. IPVH00010, Millipore Sigma, MO, USA) and blocked with 5% non-fat dry milk in 1X Tris-buffered saline with 0.1% Tween 20. Subsequently, the membranes were incubated overnight at 4°C with the respective primary antibodies. After primary antibody incubation, the membranes were treated with secondary antibodies for 1 h at room temperature. Chemiluminescence detection was performed using SuperSignal West Pico, Dura, Femto, and Atto substrates (Cat. No. Nos. 34580, 34076, 34096, A38556, Thermo Fisher Scientific, MA, USA) using an iBright750 Imager. Images were quantified using ImageJ software, and fold changes in protein expression were normalized to β-actin as an internal control. The results of each Western blot were recorded from 3-4 animals/group. Each illustration is from a representative band obtained from a single animal representative of the group.

#### Immunohistochemistry

On day two after MPTP intoxication, the animals were sacrificed, brains were removed, bisected longitudinally, and hemispheres were fixed in 4% paraformaldehyde (PFA) solution. The right hemispheres were sectioned at a thickness of 30 µm using a cryotome (CryoStar NX50; Thermo Fisher Scientific). Midbrain sections from the right hemisphere were stained with anti-CD11b antibody (1:1000) (catalog. No. MCA711, Biorad, CA, USA) to identify activated microglia. Microglial quantification was performed by counting the microglia in each section and normalizing the count to the traced area (in square millimeters). On day seven following MPTP treatment, a different cohort of mice was sacrificed to assess the number of dopaminergic neurons. After sacrifice, the mouse brains were perfused with 4% PFA. Midbrain sections containing the substantia nigra pars compacta (SNpc) and basal ganglia sections containing the striatum were stained with anti-TH (1:2000) (Cat. No. 657012, CalBiochem, EMD Millipore, MA, USA). Antibody labeling was visualized using HRP-streptavidin (ABC Elite Vector Kit; Vector Laboratories, CA, USA), followed by diaminobenzidine (DAB) color development (Sigma-Aldrich, MO, USA) in the presence of H₂O₂. Dopaminergic neuronal survival and microglial activation were quantified by a blinded investigator using the optical fractionation workflow on Stereo Investigator software (MBF Bioscience, VT, USA). Striatal dopaminergic termini were quantified using digital densitometry with ImageJ software (NIH).

#### Flow cytometry

Treg frequencies were determined from whole blood pre- and post-treatment and from the spleens at sacrifice. Splenocytes (10^6^ cells) and whole blood (~50 μL) were fluorescently labeled using PerCP-Cy5.5-anti-CD3 (Cat. number. 45-0037-42; eBioscience, CA, USA), PE-Cy7-anti-CD4 (cat. No. 25-0042-82; eBioscience, CA, USA), FITC-anti-CD8 (Cat. No. 11-0088-42, eBioscience, CA, USA) and PE-anti-CD25 (Cat. number. 12-0251-82; eBioscience, CA, USA). FoxP3 cells were fixed with 4% PFA, followed by permeabilization (FOXP3/Transcription Factor Staining Buffer Set, Cat. no. 00-5523-00, eBioscience, CA, USA) at 4 °C for 45 min. After permeabilization, the cells were labeled with APC-anti-FOXP3 (Cat. number. 17-4777-42; eBioscience, CA, USA), and stained cells were analyzed with a BD LSRII flow cytometer interfaced with FACS Diva software (BD Biosciences, San Jose, CA, USA). The total percentage of lymphocytes was used to determine the frequency of Treg cells in each sample. Following the manufacturer's instructions, CD4+CD25+ and CD4+CD25- T cells were isolated from the spleens using the EasySep Mouse CD4+CD25+ Treg Isolation Kit II (StemCell, Florida, USA). The EasySep PE Positive Selection Kit II (StemCell Technologies, Vancouver, Canada) was used to isolate CD4+CD35+ T cells through positive magnetic separation. The CD4+CD25- T cell fraction was collected from the spleens of the PBS group to serve as the Tresponder (Tresp) population in the suppression assay. Carbosyfluorescein succinidyl ester (CFSE) (Thermo Fisher, MA, USA) was used to label Tresp cells and track proliferation. For the assay, Tregs were serially diluted and co-incubated with Tresp 50 × 10^6^ cells/ mL in wells of a 96-well U-bottom microtiter plate such that Treg: Tresp ratios were 2:1, 1:1, 0.5:1, 0.25:1, and 0.125:1. Dynabeads T-activator CD3/CD28 were used at a 1:1 bead-to-cell ratio to stimulate the proliferation of Tresp cells. Stimulated and unstimulated Tregs were plated without Treg cells as controls. Cultures for the suppression assay were incubated at 37 °C in 5% CO_2_ for 3 days, after which the cells were analyzed using an LSRII flow cytometer to quantify the population of CFSE-fluorescent cells. Flow cytometry results were analyzed using the FACS DIVA Software (BD Biosciences, San Jose, CA, USA). The formula for calculating Treg-mediated inhibition was as follows: inhibition = (% proliferating Treg)/ (% Proliferating Stimulated Treg Alone).

#### Cytokine arrays

Following the manufacturer's instructions, cytokine and chemokine levels in brain lysates (Substantia Nigra and Striatum) were assessed using a mouse cytokine antibody array (catalog number: ab133995, Abcam, MA, USA). Briefly, tissues were homogenized in PBS containing a protease and phosphatase inhibitor cocktail (catalog number). No. 78440, ThermoFisher, MA, USA). Protein levels were evaluated using a bicinchoninic acid kit (catalog number: Number. 23235, Thermo Fisher, MA, USA), and the samples were diluted to a final protein concentration of 1 μg/ μL in a blocking buffer. The membranes were incubated overnight with 1 μg/ μL sample. The next day, membranes were washed and incubated with a biotinylated antibody cocktail, followed by HRP-streptavidin and visualized with DAB/H_2_O_2_. Membranes were scanned on an iBright™ CL750 Imaging System, and signal density quantification was achieved with ImageJ Launcher software (version 1.4.3.67, NIH, Bethesda, MD, USA). Brain lysates from PBS-treated mice were used as the controls.

#### RNA sequencing and transcriptomics

Flash-frozen tissues from the substantia nigra and striatum of mice (N = 3 per group) were harvested on day 2 for transcriptomic analyses. Tissues were thawed on ice and homogenized using a TissueLyser II (Qiagen, Hilden, Germany) with 5 mm stainless steel beads (cat. no. 69989; Qiagen, Hilden, Germany). Total RNA was isolated using an RNeasy Mini Kit (cat. no. 74104, Qiagen, Hilden, Germany), and RNA purity (260/280 ratio: 1.8-2.2) was evaluated using the Agilent 2100 Bioanalyzer employing the RNA 6000 Nano Kit (cat. No. 5067-1511, Agilent, CA, USA). RNA sequencing (RNA-seq) was performed on the Illumina HiSeq 2000 platform using a NovaSeq 6000 SP-200 cycle kit (paired-end, 101 bp reads) (cat. No. 0028315, Illumina). Furthermore, data preprocessing was conducted using the nf-core v3.12.0 pipeline within Nextflow v23.04.1. This involved trimming the raw fastq files using cutadapt and TrimGalore to remove sequencing adapters, terminal unknown bases (Ns), and low-quality bases at the 3' end (Phred score < 30). Quality control of the trimmed fastq files was performed with FastQC, a tool designed for high-throughput sequencing quality assessment, available online at http://www.bioinformatics.babraham.ac.UK/projects/ fastqc. The processed reads were aligned to the mm10/GRCm38 mouse reference genome using the STAR aligner, and gene-level quantification was performed using the RSEM. Differential gene expression (DEG) analysis was performed using DESeq2. P-values were corrected for multiple hypothesis testing using the Benjamini-Hochberg method to control the false-discovery rate (FDR). Genes with an absolute fold change (FC) of ≥ 1.5 and an adjusted p-value of ≤ 0.05 were classified as significantly differentially expressed. Principal component analysis (PCA) and clustering were performed using the iDep 2.0 Software Suite. Pathway enrichment and functional analyses of disease-related mechanisms were performed using Ingenuity Pathway Analysis (IPA; Qiagen).

#### Real-time qPCR

Total RNA was isolated from brain lysates (Substantia Nigra and Striatum) using the Quick RNA MicroPrep kit (R1055, Zymo Research Corporation, USA) according to the manufacturer's protocol, and RNA quantification was performed using a NanoDrop 2000 spectrophotometer (ThermoFisher Scientific, MA, USA). One μg of total RNA was used for reverse transcription 16. Primers included LCN2 (Mm01324470_m), CD68 (Mm03047343_m1), CD32 (Mm00438875_m1), CD16 (Mm00438882_m1), Msr1 (Mm00446214_m1), CD33 (Mm00491152_m1), Socs3 (Mm00545913_s1), IL1r1 (Mm00434237_m1), CCR6 (Mm99999114_s1), Chil3 (Mm00657889_mH), CCL21 (Mm03646971_gH), S100a4 (Mm00803372_g1),CCL4 (Mm00443111_m1), Arg1 (Mm00475988_m1), all from ThermoFisher Scientific, MA, USA). Five microliters of the generated cDNA was used in qPCR, along with 10 μL of TaqMan 2× Universal PCR Master Mix, 4 μL of nuclease-free water, and 1 μL of the respective 20× TM primer. mRNA expression was quantified by normalizing to GAPDH (Mm99999915_g1), and the fold change was calculated. The specificity of the RT-qPCR was controlled using a non-template control.

#### Isolation and sequencing of C-EV miRNAs

C-EV RNAs were isolated using the miRNeasy Kit (Cat no/ID: 217084, Qiagen). Small RNA sequencing was performed by LC Sciences (Houston, TX, USA) using (Illumina platform) on colostrum EV miRNAs. LC Sciences conducted small RNA library preparation, deep sequencing, and initial bioinformatics analysis, including the alignment of raw reads to miRBase and normalization of read counts. The potential functions and pathways of the genes targeted by C-EV-derived miRNAs were investigated using the PANTHER classification system.

#### miRNA C-EV loading and super-resolution imaging

C-EVs were loaded with Atto488-labeled miRNA using the Exo-Fect Exosome Transfection Kit (System Biosciences, Mountain View, CA, USA), according to the manufacturer's protocol. Following transfection, C-EVs were stained with the lipophilic fluorescent dye, PKH26, to enable membrane visualization. To ensure the removal of unincorporated dye and miRNA, the labeled C-EVs were purified by gradient ultracentrifugation. Purified C-EV samples were imaged using a Zeiss ELYRA PS.1 super-resolution microscopy system, which allowed high-resolution visualization of miRNA incorporation and C-EV morphology.

#### Murine BV2 microglial activation

BV2 microglial cells were maintained in Dulbecco's Modified Eagle Medium (DMEM) supplemented with 10% (v/v) heat-inactivated fetal bovine serum (FBS), 100 U/ mL penicillin, and 100 μg/ mL streptomycin at 37°C in a humidified 5% CO₂ atmosphere, using 75 cm² culture flasks for routine maintenance. For experimental treatments, cells were seeded at 2 × 10⁵ cells per well in 24-well plates and allowed to adhere for 24 hours. The experimental protocol consisted of first treating cells with C-EVs for 24 hours, followed by stimulation with 1 ng/mL lipopolysaccharide (LPS) for 2 hours to induce inflammatory activation. After treatment, the cells were immediately lysed using TRIzol™ Reagent (Thermo Fisher Scientific Inc.) for subsequent RNA isolation. Total RNA was extracted from BV2 cells using TRIzol™ reagent, according to the manufacturer's instructions. The isolated RNA was reverse transcribed into cDNA using the Verso cDNA Synthesis Kit (Thermo Fisher Scientific Inc.). Quantitative real-time PCR was performed on a QuantStudio™ 3 Real-Time PCR System (Thermo Fisher Scientific, Waltham, MA) using the following mouse-specific primer sets: IL-1β (forward: 5'-GTGGCAGCTACCTGTGTCTT, reverse: 5'-CTCTGCTTGTGAGGTGCTGA), TNF-α (forward: TTCACTGGAGCCTCGAATGTC, reverse: CTGTGAGGAAGGCTGTGCATT), and GAPDH (forward: AGGTTGTCTCCTGCGACTTCA, reverse: TCCACCACCCT-GTTGCTGTA) as an endogenous control.

### Statistical analysis

All grouped data are presented as mean ± standard error of the mean (SEM). Student's t-test for two groups and one-way analysis of variance (ANOVA) were used to assess statistical significance across different groups, followed by Tukey's post hoc test for multiple comparisons. Linear regression analysis was used to determine Treg function. GraphPad Prism software (version 5) was used for statistical analysis, and a p-value < 0.05 was considered statistically significant.

## Results

### C-EV characterization and biodistribution

**Figure 1:**
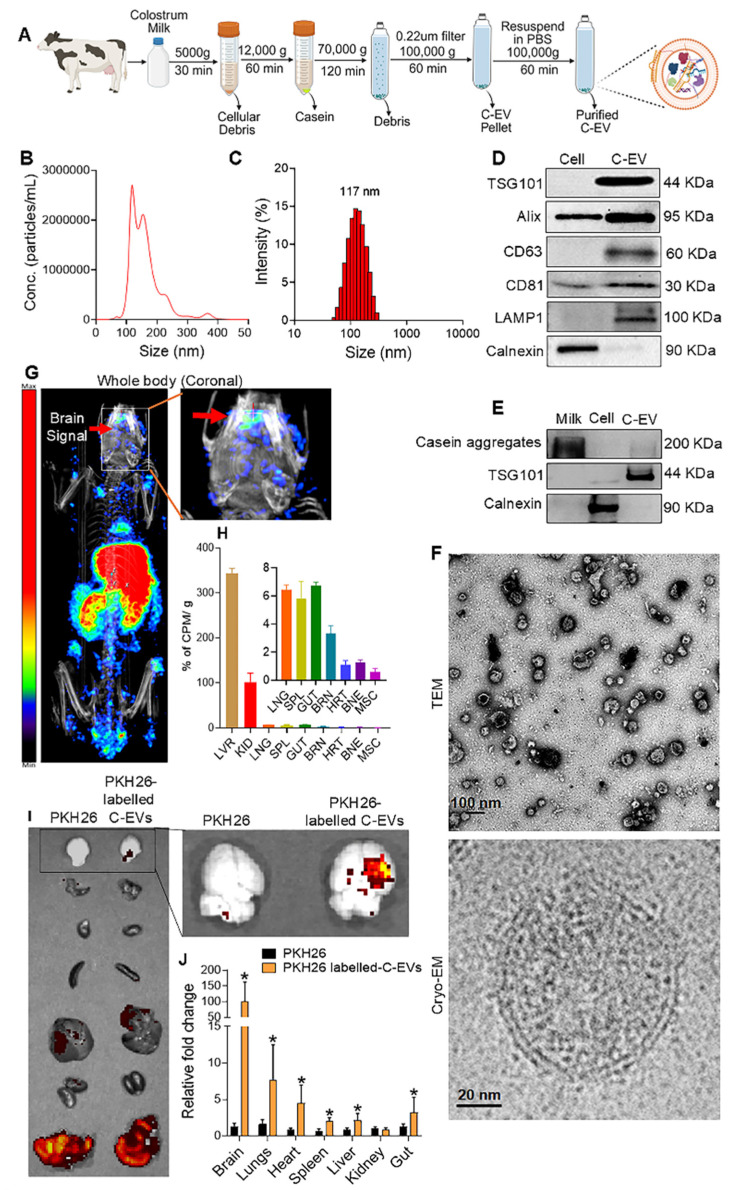
** C-EV isolation and characterization.** (A) Schematic representation of the C-EV isolation. (B) Nanoparticle tracking analysis (NTA) graph showing the particle size distribution. (C) Histogram of the size distribution obtained from dynamic light scattering. (D) Representative western blots of EV markers ALIX, TSG101, CD63, CD81, LAMP1, and the ER marker Calnexin for both C-EVs and cell lysates. (E) Representative western blots for EV marker TSG101, and Casein aggregates for C-EVs isolated by Optiprep gradient ultracentrifugation. (F) Transmission electron microscopy (scale bar: 100 nm) and cryo-electron microscopy (scale bar: 20 nm) images of C-EVs. (G) Biodistribution of ^64^Cu-labeled C-EVs by PET-CT imaging. (H) Gamma scintillation was used to quantify the relative concentration of ^64^Cu-labeled C-EVs in selected organs ex vivo (arrows indicate brain accumulation of C-EVs). (I) Representative IVIS images of PKH26-labelled C-EV distribution and quantification using Image J Launcher software (J). Data are expressed as mean ± SEM. N = 3-4/ group. Scale bar: 100, 20 nm. Abbreviations: EVs, extracellular vesicles; LVR, liver; KID, kidneys; SPL, spleen; GUT, intestine; BRN, brain; HRT, heart; BNE, bone; MSC, muscle; Cryo-EM, cryogenic electron microscopy.

Colostrum C-EVs were isolated and characterized using differential ultracentrifugation (Figure 1A). The size distribution of the purified C-EVs ranged from 50 to 150 nm, with a peak particle diameter of 121 nm (Figure 1B) 32. Dynamic light scattering (DLS) confirmed these findings, showing an average polydispersity index (PDI) of 0.21, which indicates a uniform size distribution. The average particle diameter was 117 nm (Figure 1C). Colostrum collected 24 h after a cow gave birth had 1.43 times higher C-EV concentration than that of colostrum collected three days postpartum (Table S1: milk-derived EV characterization at different time points). Therefore, C-EVs were isolated from colostrum collected 24 h after birth. Western blot analysis of C-EV lysates demonstrated the presence of exosomal markers, such as tumor susceptibility gene 101 (TSG101), ALG-2 interacting protein X (Alix), cluster of differentiation (CD)- CD63, CD81, and lysosomal-associated membrane protein (LAMP)1 (Figure 1D). To assess C-EV purity, western blot analysis using calnexin (a non-exosomal marker) was performed. To ensure that residual casein did not interfere with anti-inflammatory responses, Optiprep was employed in replicate tests 33, confirming the presence of the exosomal marker TSG101 (Figure 1E). TEM revealed a cup-shaped C-EV morphology, and cryo-EM images demonstrated the expected EV double-membrane structure (Figure 1F). Additionally, the biodistribution of the injected ^64^Cu-radiolabeled EVs was determined using CT and PET imaging. At 6 hours, C-EVs were detected in the liver, spleen, and kidney (Figure 1G) then was confirmed by *ex vivo* gamma scintillation analysis (Figure 1G,H). Gamma scintillation signals in brain tissue were higher than those in the heart, muscle, or bone (~3.5%) (Figure 1H), consistent with prior studies 21. To confirm C-EV biodistribution in C57/BL6 mice, vesicles were labelled with PKH26 and imaged using IVIS at 24h. C-EVs were distributed in the brain, lungs, heart, spleen, liver, kidney, and gut. Quantification using ImageJ Launcher software demonstrated a significant (p < 0.05) increase in the mean fluorescence intensity (PKH-26 labelled C-EVs) in the brain (Figure 1I-J).

### C-EV control of microglial activation results in neuroprotective responses

**Figure 2:**
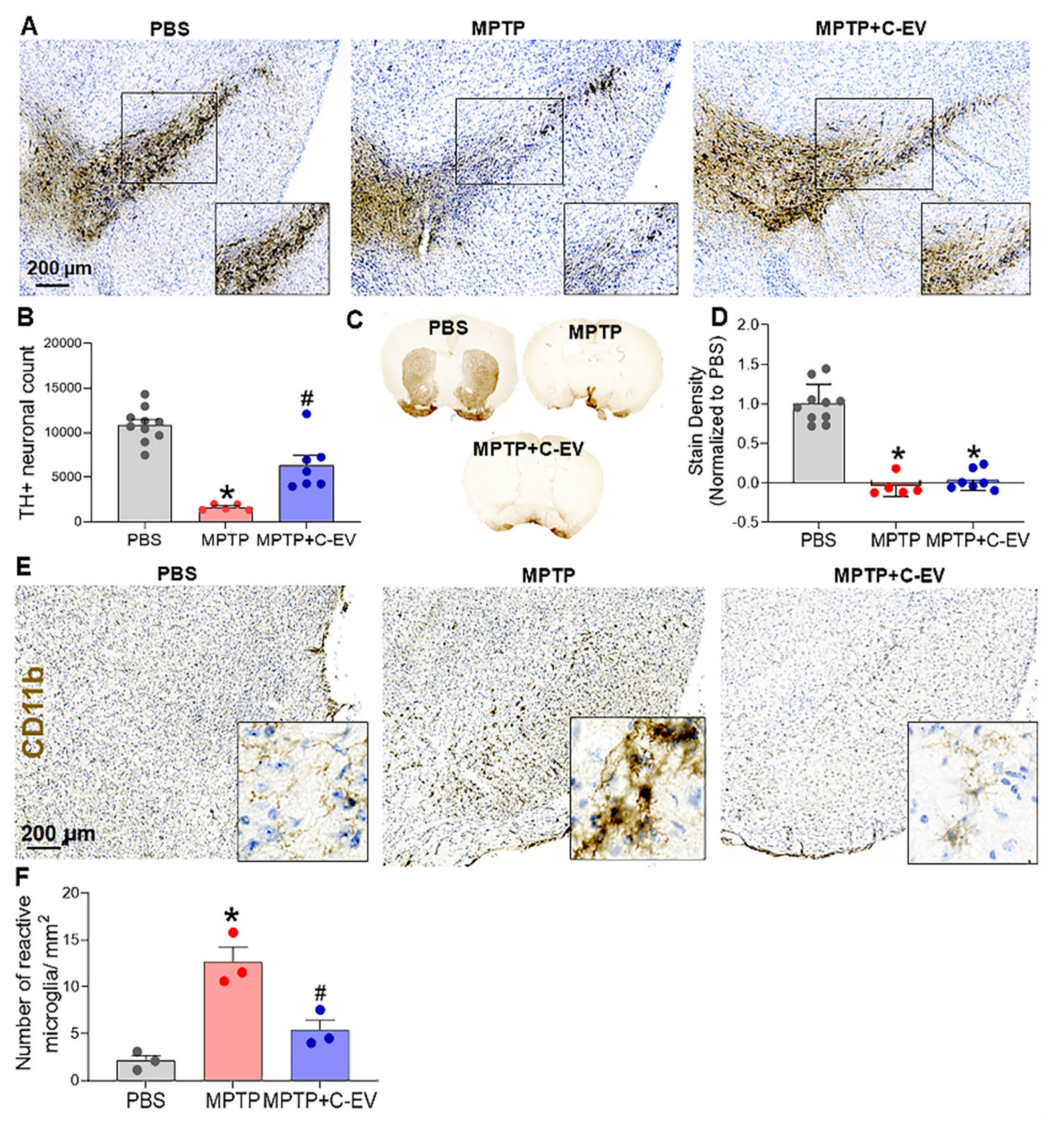
** C-EVs attenuated neurodegeneration in MPTP mice**. (A) Representative IHC images of dopaminergic neurons (TH+) in the substantia nigra (SN). (B) TH+ Neuron Count quantified using StereoInvestigator. (C) Representative IHC images of dopaminergic neurons (TH+) in the striatum of the brain. (D) ImageJ quantification of color density in the striatum. (E) Representative images of activated microglia (CD11b). (F) Quantification of activated microglia in the SN. Data are expressed as mean ± SEM. N = 10 in PBS, N = 5 in MPTP, and N = 7 in C-EV-treated MPTP mice. *p < 0.05, versus PBS; #p < 0.05, versus MPTP. Scale bar: 200 µm. Abbreviations: SN: Substantia nigra; IHC: Immunohistochemistry; PBS: Phosphate-Buffered Saline; MPTP: 1-Methyl-4-phenyl-1,2,3,6-tetrahydropyridine; C-EVs: Colostrum-derived extracellular vesicles.

MPTP-injected and C-EV-treated mice were evaluated for microglial activation and dopaminergic neuronal survival on days two and seven, respectively. C-EV treatment administered before and on the day of MPTP intoxication showed a trend toward increased survival of dopaminergic neurons in the substantia nigra (SN), although the difference was not statistically significant when compared with mice treated with MPTP alone (Figure S1A-B). Notably, there was a substantial decrease in the survival of dopaminergic neurons in MPTP-treated mice. In a subsequent study, continuous administration of C-EVs (starting three days before and ending four days after MPTP intoxication) significantly (p < 0.05) improved nigral dopaminergic neuron survival by C-EV administration that continued after MPTP (Figure 2A). Stereological analyses confirmed these results. For MPTP-intoxicated mice, there was an 86% reduction in the survival of SN dopaminergic neurons. In contrast, the C-EV-treated mice showed only a 53% reduction in neuronal count. These results indicated a 33% neuroprotective effect due to continuous C-EV treatment (Figure 2B). Striatal dopaminergic terminals in both the MPTP and MPTP + C-EV groups did not differ significantly (Figure 2C-D). Microglial activation was assessed in parallel studies by measuring CD11b+ microglial cells on day two after MPTP administration. MPTP significantly increased (p < 0.05) the number of activated amoeboid microglia to 12.66 cells/mm² from 2.11 cells/mm² in the PBS-treated group. Interestingly, C-EV administration in MPTP mice reduced the number of activated microglia to 5.37 cells/mm² (Figure 2E-F).

### PCA pathway analyses and k-clustering

**Figure 3:**
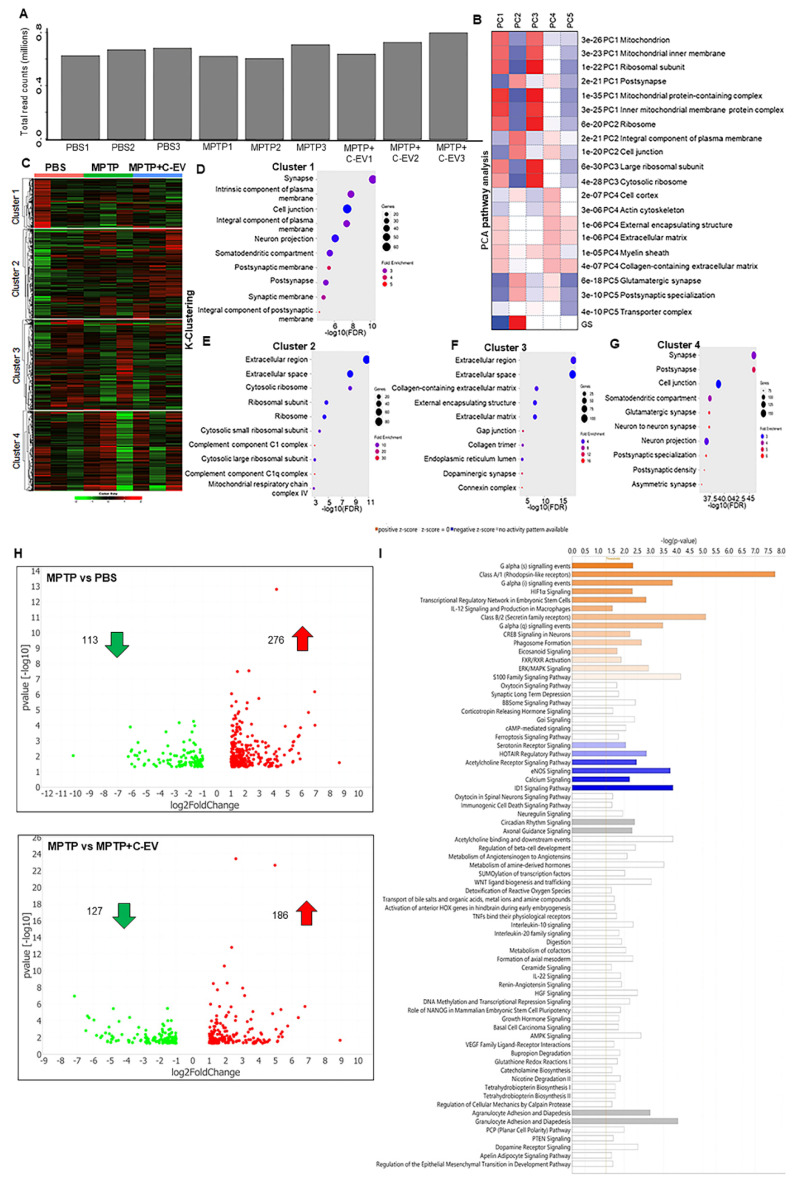
** Enrichment pathway analysis of C-EV-treated MPTP mice.** (A) Total read counts represent the number of transcripts sequenced from each group of mice used in downstream analyses. (B) PCA of the gene expression. (C) K-means clustering of gene expression. (D-G) Bubble plots of key enrichment pathways by k-means clustering. (H) Volcano plots showing the differentially expressed genes. (I) Pathway enrichment by IPA analysis. N = 3/group. Abbreviations: PBS: Phosphate-Buffered Saline; MPTP: 1-methyl-4-phenyl-1, 2, 3, 6-tetrahydropyridine; C-EVs: Colostrum Extracellular Vesicles.

To investigate the effects of C-EV treatment in MPTP-intoxicated mice, tests of differential gene expression were performed. In these experiments, the substantia nigra and striatum were isolated from mice treated with PBS (control), MPTP, or MPTP and C-EV on day two. Bulk RNA sequencing (RNA-seq) was performed on isolated transcripts. Transcripts were mapped to the mouse genome, and only annotated genes were included in the analysis, whereas non-annotated genes were excluded. The total read counts for each sample ranged from 0.6 to 0.8 million reads. To account for variations in sequencing depth and read counts between samples, the DESeq2 normalization method was applied before the differential expression analysis (Figure 3A). PCA of the normalized data revealed distinct transcriptional profiles across the different experimental groups. Genes contributing the most to the variance in principal components 1-5 (PC1-PC5) were subjected to Gene Ontology (GO) and KEGG pathway enrichment analyses. PC1 represented the largest source of variation and revealed significant differences in the expression of genes related to critical biological processes, such as mitochondrial function, mitochondrial inner membrane, ribosomal subunit, and myelin sheath pathways (Figure 3B). These findings suggest that mitochondrial dysfunction and ribosomal dysregulation are the primary outcomes that potentially contribute to neuronal damage, while the involvement of the myelin sheath indicates changes in axonal integrity and neurotransmission. PC2 revealed distinct variations in genes associated with synaptic and membrane-related pathways, including those involved in the postsynaptic membrane, glutamatergic synapses, cell junctions, and plasma membrane components. This suggests that MPTP toxicity and EV treatment affect synaptic plasticity and cell communication. PC3 cells highlighted differences in mitochondrial and ribosomal pathways, reinforcing the importance of these pathways in the treatment response. Additionally, the focus of PC3 on myelin sheath-related genes indicates the potential disruption of neuronal support and conduction, further implicating MPTP-induced neurotoxicity and potential C-EV-mediated protection. PC4 was associated with significant changes in genes related to the cell cortex, actin cytoskeleton, extracellular matrix, and collagen-containing extracellular matrix pathways. These pathways are vital for cellular structure and integrity, suggesting that treatments may influence cytoskeletal reorganization and extracellular matrix remodeling, thereby affecting cellular communication and structural support in the brain. PC5, however, displayed more complex and mixed results, with genes involved in multiple pathways showing varying expression levels but no clear single pattern. This suggests that PC5 may capture subtle yet diverse biological processes affected by the treatment.

Following PCA, k-clustering of the top 2000 genes identified four distinct clusters in PBS-, MPTP-, and MPTP/C-EV-treated mice (Figure 3C). Each cluster showed enrichment for specific Cellular Component Pathways (CCP), providing insight into the underlying biological processes affected by the treatments. Cluster 1 displayed a significant enrichment of genes associated with neuronal pathways and a notable upregulation of genes related to neuronal structure and function (Figure 3D). Cluster 2 shows the highest number of genes and fold enrichment associated with the innate immune response. Notably, there was a 30-fold enrichment of genes related to complement component C1q (Figure 3E), a key mediator of immune responses involved in neurodegenerative processes. Additionally, there was a 10-fold enrichment of genes related to the mitochondrial respiratory chain complex IV. This is particularly relevant given MPTP's known effects of MPTP on mitochondrial function. Cluster 3 exhibited a 16-fold enrichment of genes related to the dopaminergic pathway (Figure 3F), which is crucial, given the well-documented impact of MPTP on dopaminergic neurons. Furthermore, Cluster 4 displayed significant enrichment of genes associated with neuronal pathways and a notable upregulation of genes related to neuronal structure and function (Figure 3G). Following k-cluster analysis, DEG analysis revealed that 113 genes were downregulated (green dots), and 276 genes were upregulated (red dots) in the MPTP compared to the PBS group, whereas 127 genes were downregulated (green dots) and 186 genes were upregulated (red dots) in the C-EV-treated MPTP group compared to MPTP alone (Figure 3H). Overall, a larger number of differentially expressed genes was observed in the MPTP compared to the PBS group (389 genes) and in the C-EV-treated MPTP group (313 genes) than in the MPTP group. This suggests that C-EV treatment may modulate genes associated with MPTP-induced changes, reflecting its protective effect against MPTP toxicity. We used unbiased IPA pathway analysis (Figure 3I) to assess the z-score changes. These analyses demonstrated activation of G-α, HIF-1α, IL12, and CREB signaling pathways, as well as phagosome formation, eicosanoid signaling, FXR/RXR activation, ERK-MAPK signaling, and S100 Family signaling pathways, while a lower z-score indicated deactivation of HOTAIR, acetylcholine receptor, eNOS, and calcium signaling pathways in the C-EV-treated MPTP mice compared to the MPTP controls.

### Immune cell responses

The measured immune cell responses involve several steps, including chemotaxis, recruitment, and activation of immune cells, such as leukocytes, phagocytes, macrophages, and granulocytes 50-52. This study evaluated these processes in response to MPTP intoxication and C-EV therapy. The data present a statistical analysis (p-values and Z-scores) of various immune cell responses, as determined by transcriptomic analysis. MPTP treatment caused a significant activation of inflammatory pathways, indicated by positive Z-scores and low p-values for immune cell responses, including phagocyte chemotaxis, leukocyte recruitment and activation, and granulocyte infiltration. The increase in these pathways suggests that MPTP triggers an active inflammatory response characterized by immune cell migration and activation.

**Figure 4:**
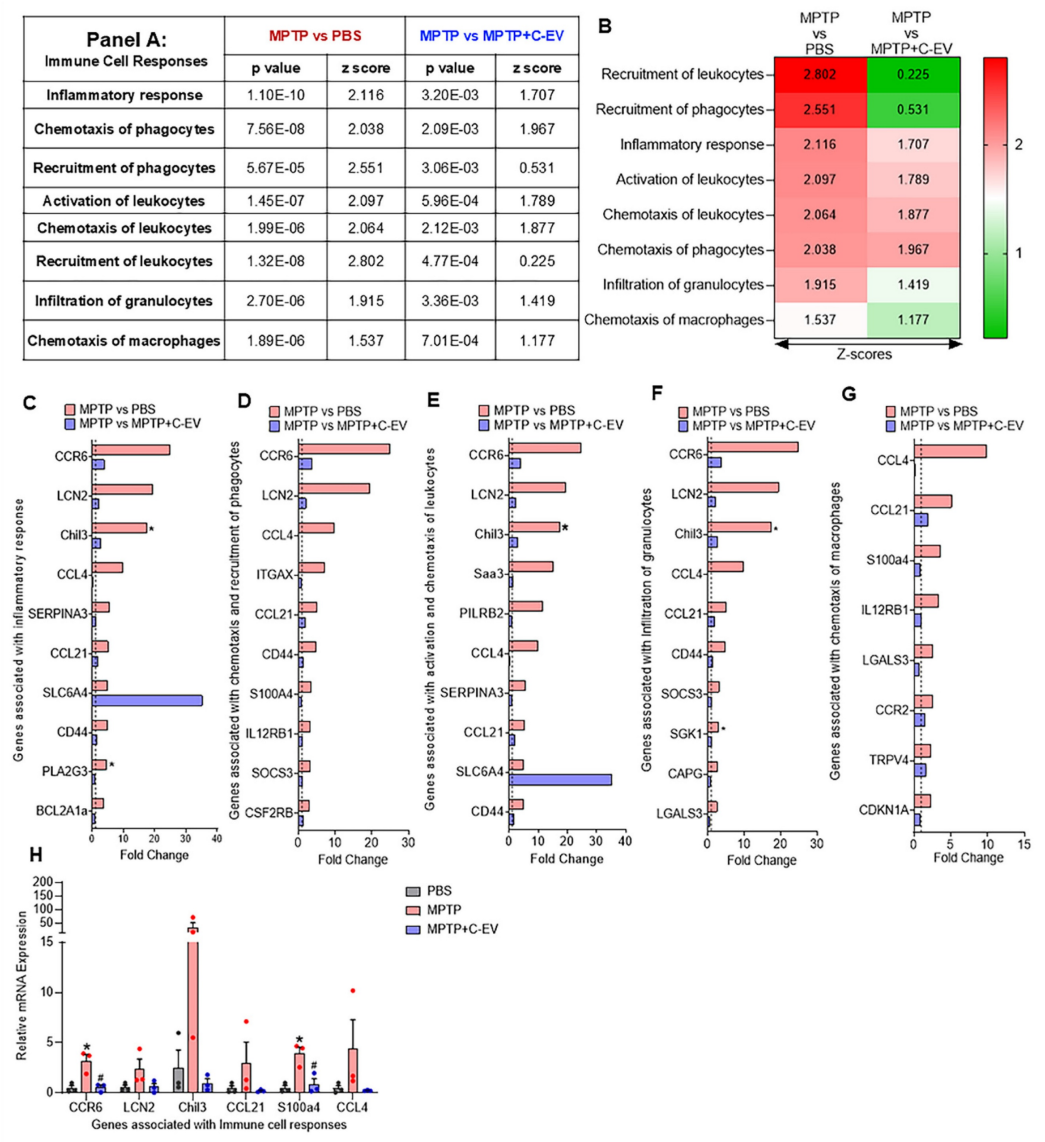
**Immune responses in C-EV-treated MPTP mice.** Panel A summarizes the z-scores and p-values of immune cell responses across the different treatment groups. (B) Differential gene expression associated with the inflammatory response. (C) Differential gene expression associated with chemotaxis and phagocyte recruitment. (D) Differential gene expression associated with leukocyte activation and chemotaxis. (E) Differential gene expression associated with granulocyte infiltration. (F) Differential gene expression associated with macrophage chemotaxis. (G) qPCR confirmatory analysis was performed. Data are expressed as fold change. The dotted line indicates the PBS group. N = 3 mice/group. *p < 0.05, versus PBS; ^#^p < 0.05, Abbreviations: *BCL2A1a*: BCL2 Related Protein A1 Alpha; *CAPG*: Capping Actin Protein, Gelsolin-Like; *CCL21*: Chemokine Ligand 21b; *CCL4*: Chemokine Ligand 4; *CCR2/6*: C-C Motif Chemokine Receptor 2/6; *CD44*: Cluster of Differentiation 44; *CDKN1A*: Cyclin Dependent Kinase Inhibitor 1A; *Chil3*: Chitinase-Like 3; *CSF2RB*: Colony Stimulating Factor 2 Receptor Beta; *IL12RB1*: Interleukin 12 Receptor Subunit Beta 1; *ITGAX*: Integrin Subunit Alpha X; *LCN2*: Lipocalin 2; *LGALS3*: Galectin-3; *PILRB2*: Paired Immunoglobulin-Like Type 2 Receptor Beta 2; *PLA2G3*: Phospholipase A2 Group III; *S100A4*: S100 Calcium Binding Protein A4; *Saa3*: Serum Amyloid A3; *SERPINA3*: Serpin Family A Member 3; *SGK1*: Serum/Glucocorticoid Regulated Kinase 1; *SLC6A4*: Solute Carrier Family 6 Member 4; *SOCS3*: Suppressor of Cytokine Signaling 3; *TRPV4*: Transient Receptor Potential Cation Channel Subfamily V Member 4.

Conversely, C-EV treatment showed a trend of regulating responses compared to MPTP mice. This was shown by reduced Z-scores, indicating anti-inflammatory responses (Figure 4A, Figure 4B). Transcriptomic analysis revealed significant changes in gene expression related to the inflammatory response, chemotaxis, recruitment, and activation of immune cells after MPTP treatment. A total of 37 genes associated with the inflammatory response were upregulated in the MPTP-treated group compared to the PBS controls, with four significantly upregulated (p < 0.05). The top 10 hit genes included *CCR6* (C-C motif chemokine receptor 6), *LCN2* (lipocalin 2), *Chil3* (chitinase-like 3), *CCL4* (C-C motif chemokine ligand 4), *SERPINA3* (Serpin family A member 3), *CCL21* (C-C motif chemokine ligand 21), *SLC6A4* (solute carrier family 6 member 4), *CD44* (CD44 molecule), *PLA2G3* (Phospholipase A2 group III), and *BCL2A1a* (B cell leukemia/lymphoma 2-related protein A1a) (Figure 4C). Among these, *Chil3* and *PLA2G3* were significantly upregulated (p < 0.05) in the MPTP group compared to the PBS group. Nine of the ten genes showed a decreasing trend in the MPTP+C-EV group compared to the MPTP group, but none were statistically significant. Additionally, *SLC6A4* was upregulated in the C-EV-treated MPTP group compared to that in the MPTP-alone group. The additional inflammation-related genes are shown in Figure S3A. Among these, *GGT5* (Gamma-glutamyltransferase 5) and Von Willebrand factor (*VWF*) were significantly upregulated (p < 0.05) in the MPTP group compared to the PBS controls. The top 10 genes included *CCR6*, which facilitates Th17 cell recruitment to inflammatory sites by interacting with *CCL20* (C-C motif chemokine ligand 20) 53. *LCN2* 54, *CCL4* 55, and *CCL21* 32 contribute to the inflammatory response. *Chil3* is a marker of M2 anti-inflammatory microglia and is increased during neuroinflammatory conditions 56. *SERPINA3*, which is mainly expressed by reactive astrocytes and microglia, modulates neuroinflammation 57. *SLC6A4* influences inflammation by regulating microglial activation and cytokine expression 58. *CD44* mediates neuroinflammation by activating microglia and macrophages 59. *PLA2G3* boosts inflammatory signaling by generating lysophospholipids in extracellular vesicles, which are converted into lysophosphatidic acid by autotaxin, a lipid mediator that amplifies inflammation 60. *BCL2A1a* influences inflammation by modulating the NF-κB and STAT pathways 61. Furthermore, some genes, such as *BCL2A1a* and *PLA2G3*, showed significant differences (p < 0.05) in C-EV-treated MPTP mice compared to PBS controls. Genes linked to chemotaxis and phagocyte recruitment included 26 upregulated genes in the MPTP group compared with the PBS group, with four significantly upregulated (p < 0.05). The top 10 genes were *CCR6, LCN2, CCL4, ITGAX* (integrin alpha X), *CCL21, CD44, S100A4, IL12RB1, SOCS3*, and *CSF2RB* (Figure 4D). Additional related genes are shown in Figure S3B. A downward trend in C-EV-treated MPTP mice was shown 62, 63, The functions [64-66] [67] [68] of the top- 10 genes involve immune cell entry to sites of inflammation sites and modulating immune responses. These responses include *CCR6* and *LCN2* directed leukocyte chemotaxis 69-72 recruitment 73, and activation 74-78. No significant differences were observed between the PBS- and EV-treated MPTP groups. Genes associated with leukocyte activation and chemotaxis (*CCR6, LCN2, SAA3, PILRB2, CCL4, SERPINA3, CCL21, SLC6A4*, and *Chil3*, p < 0.05) were upregulated in MPTP mice, whereas the C-EV-treated mice showed downregulation, with the exception of *SLC6A4*. Figure 4F shows the genes involved in cell infiltration. Among the 19 upregulated genes, three were significantly increased (p < 0.05) in the MPTP group compared to the PBS group. The top hits included *CCR6, LCN2, Chil3, CCL4, CCL21, CD44, SOCS3, SGK1, CAPG*, and *LGALS3*, with *Chil3* and *SGK1* being significantly upregulated (p < 0.05). Figure S3D illustrates additional gene profiling. Despite a decreasing trend in the C-EV-treated MPTP mouse group, none reached significance. 79. These genes signal neutrophil recruitment, tissue infiltration, inflammation, activation, and function 80. Figure 4G shows the genes involved in myeloid cell chemotaxis: among the eight upregulated genes 81,68, none were significantly increased in the MPTP group compared to the PBS group 82. These genes, including *CCL4, CCL21, S100A4, IL12RB1, LGALS3, CCR2, TRPV4*, and *CDKN1A*, are associated with immune cell recruitment 83, activation 84, and response modulation 85. Collectively, these gene changes highlight aspects of immune cell recruitment, activation, and inflammatory responses induced by MPTP and suggest a protective role for EVs in these processes. It is also noted that, despite a downregulated gene expression trend in the C-EV-treated MPTP mice relative to the MPTP mice (*PLA2G3, ITGAX,* and* CDKN1A*) were upregulated.

Compared to MPTP, C-EV-treated MPTP mice demonstrated a downregulated inflammatory gene profile. Of the 37 upregulated pro-inflammatory genes, C-EV treatment downregulated 34. This reduction supports the notion that C-EVs have a protective role during MPTP nigral degeneration by reducing pro-inflammatory pathways and the recruitment and activation of neurodegenerative immune cells at the disease sites. Among the genes associated with chemotaxis, 24/28 genes showed reduced expression in the C-EV group. All genes involved in macrophage chemotaxis showed a trend of downregulation after EV treatment. These data highlight the ability of C-EVs to reduce macrophage recruitment to sites of neuroinflammation. These results suggest that C-EVs suppress MPTP-induced inflammatory processes. This process affects the recruitment, chemotaxis, and activation pathways of immune cells. Interestingly, *BCL2A1d* (B cell leukemia/lymphoma 2 related protein A1d), *ESR1* (estrogen receptor 1), and *SL6A4* (solute carrier family 6 member 4) are associated with inflammatory response; *PRKCD* (protein kinase C delta) gene is associated with chemotaxis and recruitment of phagocytes; *SL6A4, Saa3*, and *PILRB2* genes are related to the activation and chemotaxis of leukocytes; and VWF, associated with the infiltration of granulocytes, were highly expressed upon C-EV treatment in MPTP mice, indicating compensatory mechanisms. Next, we validated the top three genes from each pathway using qPCR (Figure 4G). The results demonstrated that *CCR6* and *S100a4* were significantly increased (p < 0.05), while *LCN2, Chil3, CCL21*, and *CCL4* showed an increasing trend in MPTP mice compared to that in PBS controls. Both *CCR6* and *S100a4* were significantly downregulated in the C-EV-treated group, which paralleled the RNA-seq datasets.

### Microglial immunity

**Figure 5:**
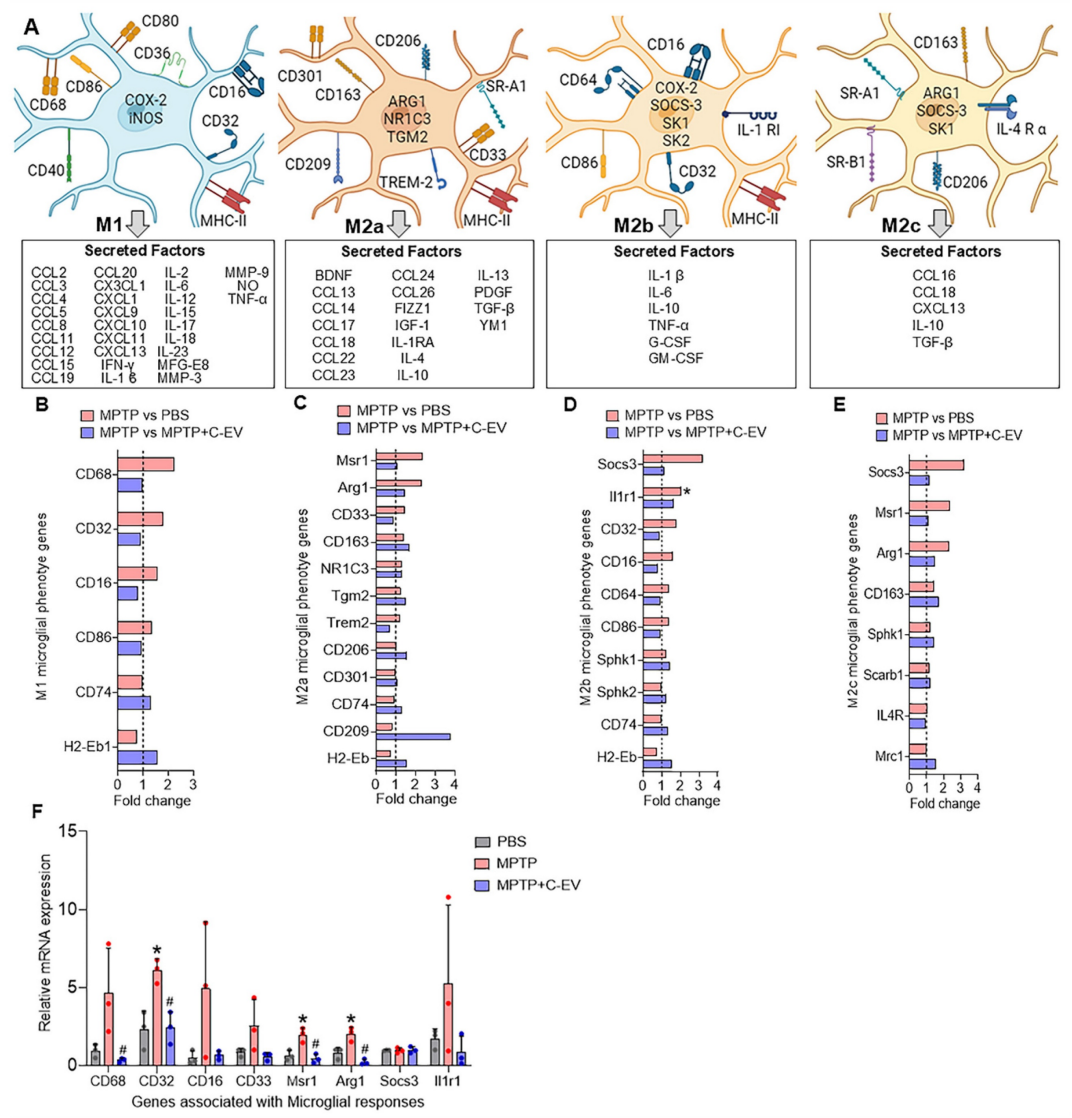
** Microglial phenotypes in C-EV-treated MPTP mice.** (A) Schematic representation of microglial phenotypes: M1 (classically activated), M2a (alternatively activated), M2b (immune regulatory), and M2c (deactivated). The M1 phenotype is associated with pro-inflammatory markers (e.g., CD68, CD80, and CD32) and cytokines such as IL-1β, TNF-α, and IL-6. The M2a phenotype is characterized by anti-inflammatory markers (e.g., CD163 and CD206) and factors such as IL-4, IL-10, and TGF-β. M2b microglia exhibit a combination of proinflammatory and anti-inflammatory properties. The M2c phenotype is characterized by deactivation and anti-inflammatory response. (B) Differential gene expression associated with the M1 phenotype. (C) Differential gene expression related to the M2a phenotype. (D) Differential gene expression related to the M2b phenotype. (E) Differential gene expression related to the M2c phenotype. Data are expressed as fold change. (F) Gene expression was analyzed using qPCR. The dotted line indicates the PBS group. N = 3 mice per group. *p < 0.05, versus PBS; ^#^p < 0.05, versus MPTP. Abbreviations: PBS: Phosphate-Buffered Saline; MPTP: 1-Methyl-4-phenyl-1,2,3,6-tetrahydropyridine; C-EVs: Colostrum Derived Extracellular Vesicles; M1, M2a, M2b, M2c: Microglial activation phenotypes; *CD68*: Cluster of Differentiation 68; *CD32*: Fc Fragment of IgG, Low Affinity IIa, Receptor (FcγRII); *CD16*: Fc Gamma Receptor IIIa (Fcgr3); *CD86*: Cluster of Differentiation 86; *CD74*: Cluster of Differentiation 74 (Invariant chain); H2-Eb1: MHC Class II, E Beta; Msr1: Macrophage Scavenger Receptor 1; Arg1: Arginase 1; *CD33*: sialic acid-binding immunoglobulin-like lectin (Siglec-3); *CD163*: Cluster of Differentiation 163; *NR1C3*: Peroxisome proliferator-activated receptor gamma (PPAR-γ); *TGM2*: Transglutaminase 2; *TREM2*: Triggering Receptor Expressed on Myeloid Cells 2; *CD206*: Cluster of Differentiation 206 (Macrophage Mannose Receptor 1); *CD301*: C-Type Lectin Domain Family 10, Member A; *CD209*: Dendritic cell-specific ICAM 3-grabbing nonintegrin (DC-SIGN); *Socs3*: Suppressor of Cytokine Signaling 3; *IL1r1*: Interleukin 1 Receptor, Type 1; *CD64*: Cluster of Differentiation 64 (Fc-gamma receptor 1;FcγRII); *Sphk1/Sphk2*: Sphingosine Kinase 1/2; *IL4R*: Interleukin 4 Receptor; *Scarb1*: Scavenger Receptor Class B Member 1; *Mrc1*: Mannose Receptor, C Type 1.

Next, we evaluated microglial activation in MPTP- and C-EV-treated mice using RNA-Seq. Four primary states of microglial activation are defined by biomarkers and secreted factors with pro-inflammatory and anti-inflammatory responses (Figure 5A). Of 6 genes associated with an M1 microglial phenotype, 4 [*CD68, CD32 (Fcgr2b), CD16 (Fcgr3), and CD86*] were upregulated in MPTP mice and showed a trend of downregulation in C-EV-treated MPTP mice compared to MPTP, however, *CD74* (Cluster of Differentiation 74 (Invariant chain) and *H2-Eb1* (MHC Class II, E Beta) showed a trend of further upregulation in the C-EV treated MPTP mice compared to MPTP alone. None of the M1 genes showed significant differences between the C-EV-treated MPTP mice and the PBS groups (Figure 5B). While 7/12 genes [*Msr1*(Macrophage Scavenger Receptor 1), *Arg1* (Arginase 1), *CD33* (CD33 molecule), *CD163* (Cluster of Differentiation 163), *NR1C3*, Peroxisome proliferator-activated receptor gamma (PPAR-γ), *TGM2* (Transglutaminase 2), *TREM2* (Triggering Receptor Expressed on Myeloid Cells 2) associated with M2a microglial phenotypes showed a trend of upregulation in MPTP compared to the PBS group, 4- (*Msr1, Arg1, CD33, TREM2*) of them showed a trend of downregulation, while 3- (*CD163, NR1C3, TGM2*) remained unchanged in C-EV treated MPTP intoxicated mice. Additionally, 3/12 genes [*CD206* (Cluster of Differentiation 206), *CD301* (C-Type Lectin Domain Family 10, Member A), and *CD74*] were unchanged, and 2/12 (*CD209, H2-Eb1*) showed a trend of downregulation in MPTP mice compared to PBS. Interestingly, *CD209* expression was further increased in the MPTP+C-EV group compared to that in the MPTP group. None of the M2a genes showed significant differences between the MPTP+C-EV and PBS groups (Figure 5C). In the microglial-M2b phenotype, 6/10 genes [*Socs3, IL1r1* (Interleukin 1 Receptor, Type 1), *CD32, CD16, CD64* (Cluster of Differentiation 64), *CD86* (cluster of differentiation 86)] showed a trend of upregulation in MPTP mice compared to PBS mice, with IL1r1 showing significant upregulation (p < 0.05). Furthermore, these genes showed a trend of downregulation in C-EV-treated MPTP mice compared to that in the MPTP mice. However, *Sphk1* (Sphingosine Kinase 1), *Sphk2* (Sphingosine Kinase 2), *CD74*, and *H2-Eb1* were further upregulated in C-EV-treated MPTP mice. Additionally, Sphk2 levels were significantly different (P < 0.05) between PBS- and C-EV-treated MPTP mice (Figure 5D). Furthermore, in the M2c microglial phenotype, of 4/8 genes (*Socs3, Msr1, Arg1,* and* CD163*) were upregulated in the MPTP group, whereas only *Socs3, Msr1,* and* Arg1* showed a trend of downregulation in the C-EV-treated MPTP mouse group. *Sphk1, Scarb1*(Scavenger Receptor Class B Member 1), and *IL4R* (interleukin 4 receptor) remained unchanged in the MPTP and MPTP+C-EV groups, whereas *Mrc1* (Mannose Receptor, C Type 1) showed an upregulation trend in the MPTP+C-EV group compared to that in the MPTP group. None of the M2c genes showed significant differences between the MPTP+C-EV and phosphate-buffered saline (PBS) groups (Figure 5E). These results demonstrate that MPTP mice exhibit both M1 and M2 microglial phenotypes. Furthermore, treatment with C-EVs showed a trend of downregulation in the expression profile of proinflammatory genes, thereby facilitating alternative microglial activation. We validated top 3- genes from each of the pathways by qPCR analysis (Figure 5F), and the results demonstrated that *CD68, CD16, CD33* and* Il1r1* showed a trend of increase, while *CD32, Arg1* and* Msr1* demonstrated a significant increase (p < 0.05) in the MPTP group compared to PBS, while C-EV-treated MPTP demonstrated significant (p < 0.05) downregulation in *CD68, CD32, Msr1* and* Arg1*, while a trend of downregulation was observed in *CD16* and* Il1r1*, compared to the MPTP group that paralleled the RNA seq data. However, Socs3 gene expression remained unchanged among the groups.

### Inflammasomes

**Figure 6:**
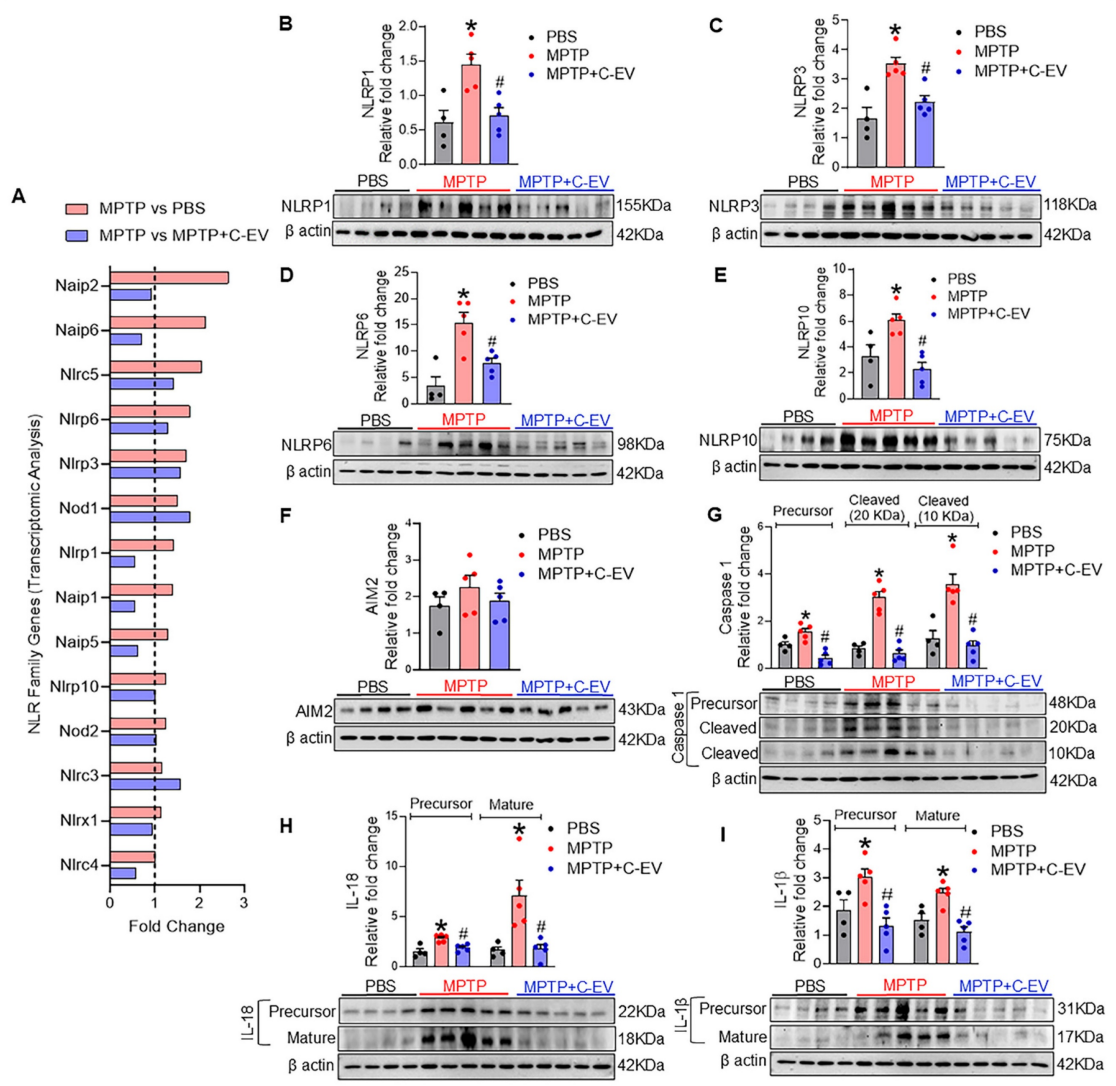
** NLR and inflammasomes in C-EV-treated MPTP mice.** (A) Differential gene expression of NLR family members. Representative Western blot images showing the expression of NLRP1 (B), NLRP3 (C), NLRP6 (D), NLRP10 (E), AIM2 (F), Caspase1 (both precursor and mature) (G), IL-18 (both precursor and mature) (H), and IL-1β (both precursor and mature) (I). Data are expressed as mean ± SEM. The dotted line indicates the PBS group. N = 4 in PBS, N = 5 in MPTP, and N = 5 in C-EV-treated MPTP. *p < 0.05, versus PBS; #p < 0.05, versus MPTP. Abbreviations: PBS: Phosphate-Buffered Saline; MPTP: 1-Methyl-4-phenyl-1,2,3,6-tetrahydropyridine; C-EVs: Colostrum Derived Extracellular Vesicles; NLRP1: NOD-Like Receptor Family Pyrin Domain Containing 1; NLRP3: NOD-Like Receptor Family Pyrin Domain Containing 3; NLRP10: NOD-Like Receptor Family Pyrin Domain Containing 10; AIM2: Absent in Melanoma 2; Caspase-1: Cysteine-aspartic protease in inflammasome activation; IL-1β: Interleukin 1 Beta; IL-18: Interleukin 18; β actin: Beta Actin, loading control; Naip: NLR Family Apoptosis Inhibitory Protein; Nod: Nucleotide-binding Oligomerization Domain-containing protein; Nlrc: NOD-Like Receptor C.

Next, we evaluated the effects of C-EVs on inflammasome expression and the modulation of neuroinflammation. Transcriptomic analysis revealed the differential expression of NLR family genes in response to MPTP treatment (Figure 6A). Several NLR genes, including *Naip2, Naip6, NLRC5, Nlrp6, Nlrp3, Nod1, Nlrp1, Naip1, Naip5, Nlrp10,* and* Nod2*, showed a trend of upregulation in MPTP compared to PBS, while treatment with C-EVs showed a trend of downregulation in the expression of *Naip2, Naip6, NLRC5, Nlrp6, Nlrp1, Naip1, Naip5, Nlrp10, Nod2, Nlrx1,* and* Nlrc4*, compared to MPTP group, suggesting that C-EVs may modulate inflammasome activation and reduce neuroinflammation. Furthermore, western blot analysis validated the transcriptomic findings by assessing the protein levels of inflammasome components (Figure 6B-I). NLRP1, NLRP3, NLRP6, and NLRP10 protein levels were significantly (p < 0.05) upregulated in the MPTP group compared with the PBS controls, indicating inflammasome activity (p < 0.05). AIM2, on the other hand, did not show significant changes in protein expression following MPTP treatment. The activation of downstream inflammasome markers, including precursor and cleaved caspase-1 and mature forms of IL-1β and IL-18, was also significantly increased (p < 0.05) in the MPTP group, indicating an active inflammatory response. Notably, C-EV treatment significantly decreased (p < 0.05) inflammasome levels, as well as precursor and cleaved caspase-1, IL-1β, and IL-18 levels. This finding supports the role of C-EVs in mitigating inflammasome-mediated inflammation.

### Cytokines and chemokines

**Figure 7:**
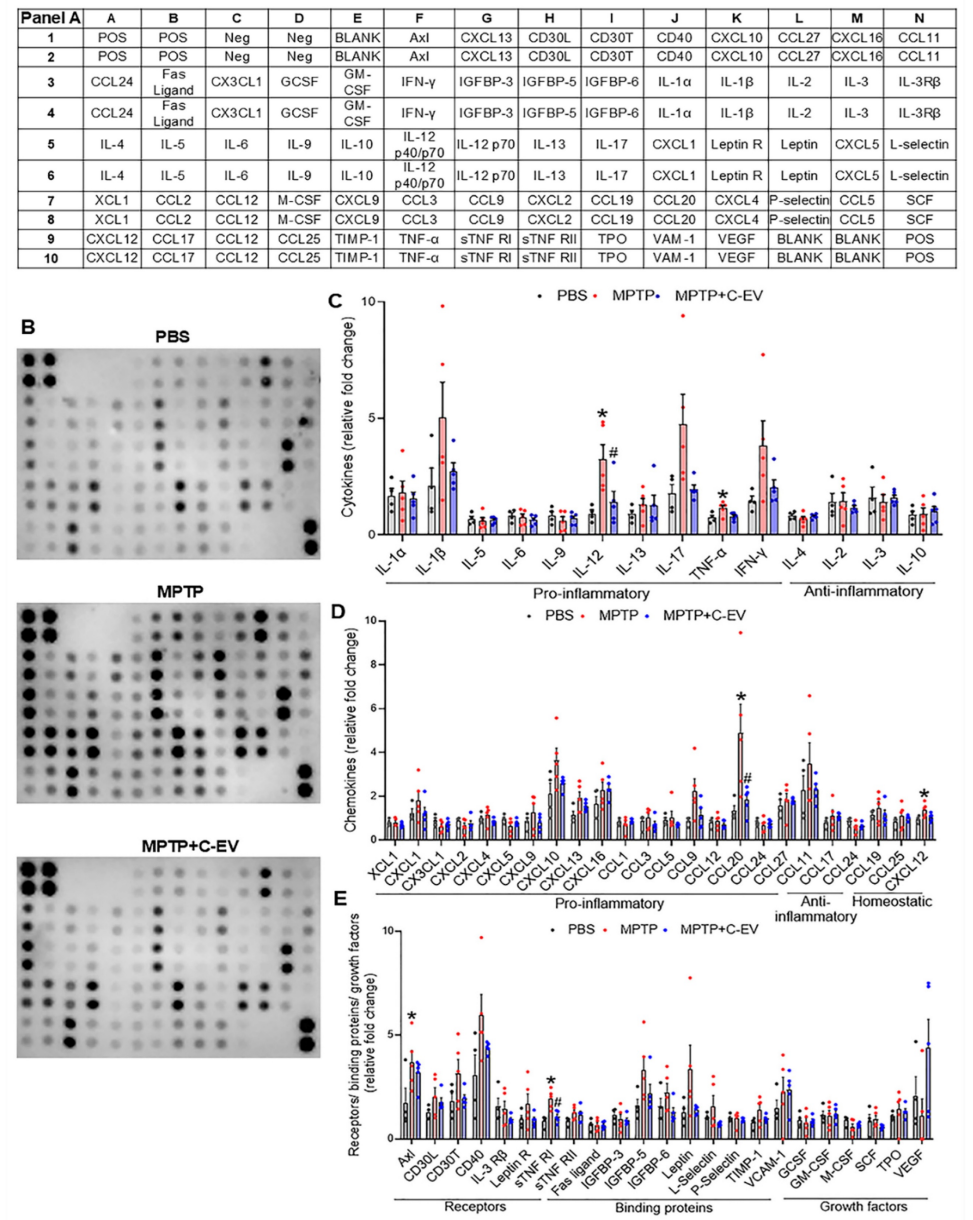
** Cytokines and chemokines in C-EV-treated MPTP mice.** Panel A shows the list of cytokines and chemokines on the array map. Panel B shows representative array blot images from the PBS, MPTP, and MPTP+C-EV groups. Quantification of cytokines (C), chemokines (D), receptors, binding proteins, and growth factors (E) in the substantia nigra and striatum of the different groups. Data are expressed as mean ± SEM. N = 4 in PBS, N = 5 in MPTP, and N = 5 in C-EV-treated MPTP. *p≤0.05 PBS vs MPTP and #p≤0.05 MPTP vs MPTP and C-EVs. Abbreviations: BCL (CXCL13); CRG-2(CXCL10); CTACK(CCL27); Eotaxin (CCL11); Eotaxin-2(CCL24); Fractalkine (CX3CL1); KC(CXCL1); LIX(CXCL5); Lymphotactin (XCL1); MCP1 (CCL2); MCP5 (CCL12); MIG(CXCL9); MIP-1α(CCL3); MIP-1γ(CCL9); MIP-2 (CXCL2); MIP-3 β (CCL19); MIP-3 α (CCL20); PF-4 (CXCL4); RANTES (CCL5); SDF-1α(CXCL12); TARC (CCL17); TCA-3 (CCL1); TECK (CCL25); IL 12 p40/p70 (IL-12).

As cytokines and chemokines play pivotal roles at the site of inflammation, we assessed their expression using a 62-cytokine/chemokine array assay (Figure 7). We prepared representative membranes from the nigra and striatum of the treated mice (Figure 7B). MPTP induced significant increases (p < 0.05) in IL-12 and TNF-α, with decreased trends in IL-1β, IL-17, and interferon gamma (IFN-γ). C-EVs significantly decreased IL-12 levels, with a decrease in IL-1β, IL-17, and IFN-γ levels (Figure 7C). For chemokines, MPTP increased (p < 0.05) CCL20 compared to PBS, whereas C-EV treatment of MPTP mice reduced CCL20 levels (Figure 7D). Similarly, the chemokine CXCL12 was significantly upregulated (p < 0.05) in MPTP mice compared to that in PBS controls. MPTP intoxication significantly upregulated (p < 0.05) inflammation-associated receptors Axl and sTNFRI, while C-EV treatment of MPTP mice significantly reduced (p < 0.05) sTNFRI expression (Figure 7E). Neither MPTP nor C-EV treatment had any significant effect on the binding proteins or growth factors.

### Neurodegeneration

**Figure 8:**
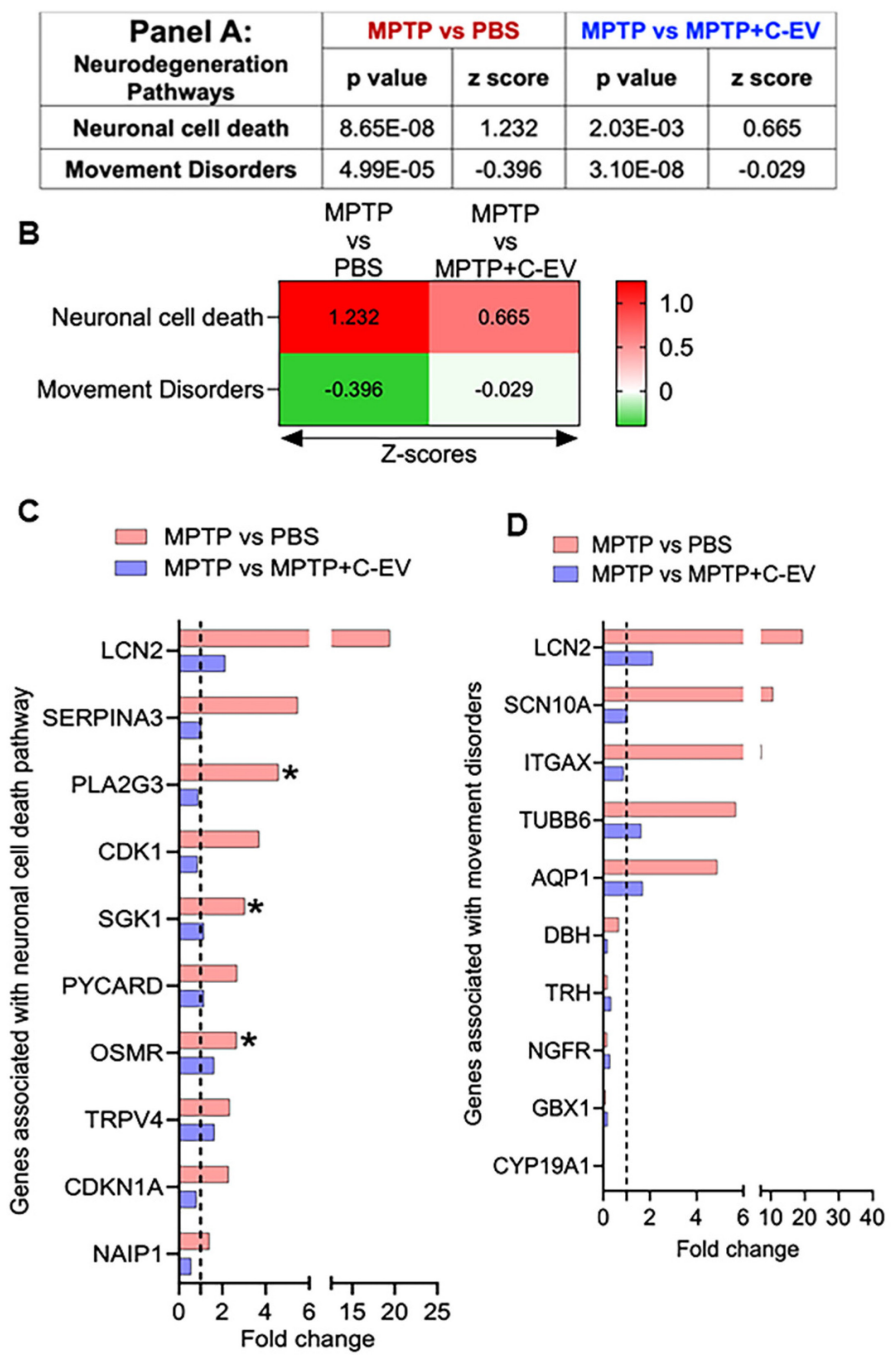
** Differentially expressed neuronal genes in C-EV-treated MPTP mice.** Panel A shows the P-values and Z-scores for genes associated with neuronal cell death and movement disorder pathways in different groups. (B) Heatmap with Z-scores. (C) Differential expression of genes involved in the neuronal cell death pathway. (D) Differential expression of genes associated with pathways involved in the movement disorders. Data are expressed as fold change. The dotted line indicates the PBS-treated group. N = 3 mice/group. *p < 0.05, versus PBS; ^#^p < 0.05, versus MPTP. Abbreviations: PBS: Phosphate-Buffered Saline; MPTP: 1-Methyl-4-phenyl-1;2;3;6-tetrahydropyridine; C-EVs: Colostrum Derived Extracellular Vesicles; *LCN2*: Lipocalin 2; *SERPINA3*: Serpin Family A Member 3; *PLA2G3*: Phospholipase A2 Group III; *CDK1*: Cyclin Dependent Kinase 1; *SGK1*: Serum/Glucocorticoid Regulated Kinase 1; *PYCARD*: PYD And CARD Domain Containing; *OSMR*: Oncostatin M Receptor; *TRPV4*: Transient Receptor Potential Cation Channel Subfamily V Member 4; *CDKN1A*: Cyclin Dependent Kinase Inhibitor 1A; *NAIP1*: NLR Family Apoptosis Inhibitory Protein 1; *SCN10A*: Sodium Voltage-Gated Channel Alpha Subunit 10; *ITGAX*: Integrin Subunit Alpha X; *TUBB6*: Tubulin Beta 6 Class V; *AQP1*: Aquaporin 1; *DBH*: Dopamine Beta-Hydroxylase; *TRH*: Thyrotropin Releasing Hormone; *NGFR*: Nerve growth factor receptor; *GBX1*: Gastrulation Brain Homeobox 1; *CYP19A1*: Cytochrome p450 family 19 subfamily a member of 1.

**Figure 9:**
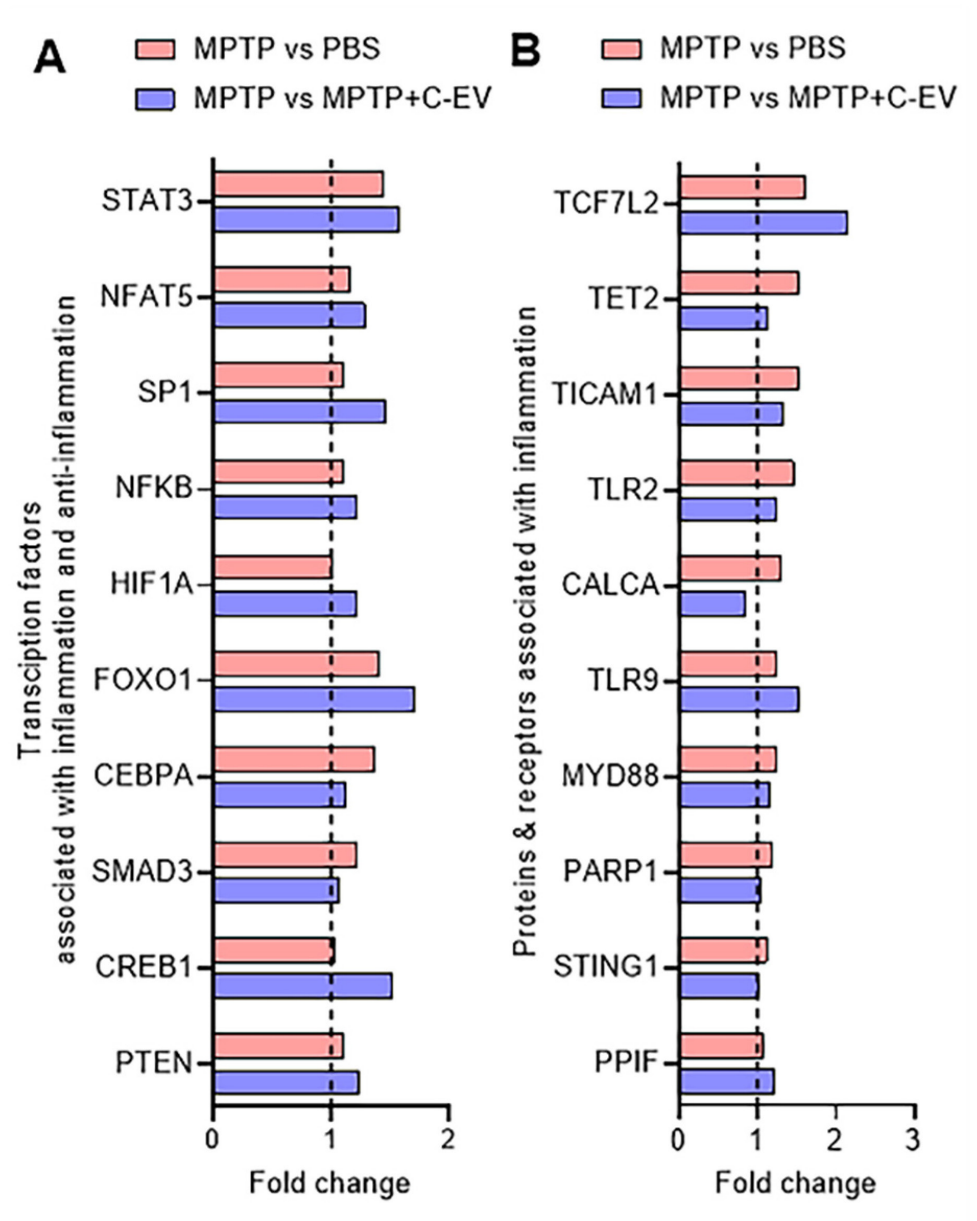
** Differentially expressed regulatory genes in C-EV-treated MPTP mice.** (A) Differential expression of key transcription factors associated with inflammation. (B) Differential expression of proteins and receptors associated with inflammation. Data are expressed as fold change. The dotted line indicates the PBS group. N = 3 mice per group. *p < 0.05, versus PBS; ^#^p < 0.05, versus MPTP. Abbreviations: PBS: Phosphate-Buffered Saline; MPTP: 1-Methyl-4-phenyl-1;2;3;6-tetrahydropyridine; C-EVs: Colostrum Derived Extracellular Vesicles; *STAT3*: Signal Transducer and Activator of Transcription 3; *NFAT5*: Nuclear Factor Of Activated T-Cells 5; *SP1*: Sp1 Transcription Factor; *NFKB*: Nuclear Factor Kappa B; ; *HIF1A*: Hypoxia Inducible Factor 1 Alpha Subunit; *FOXO1*: Forkhead Box O1; *CEBPA*: CCAAT Enhancer Binding Protein Alpha; *SMAD3*: SMAD Family Member 3; *CREB1*: cAMP Responsive Element Binding Protein 1; *PTEN*: Phosphatase And Tensin Homolog; *TCF7L2*: Transcription Factor 7 Like 2; *TET2*: Tet Methylcytosine Dioxygenase 2; *TICAM1*: Toll-Like Receptor Adaptor Molecule 1; *TLR2*: Toll-Like Receptor 2; *CALCA*: Calcitonin-Related Polypeptide Alpha; *TLR9*: Toll-Like Receptor 9; *MYD88*: Myeloid Differentiation Primary Response 88; *PARP1*: Poly(ADP-Ribose) Polymerase 1; *STING1*: Stimulator Of Interferon Response CGAMP Interactor 1; *PPIF*: Peptidylprolyl Isomerase F.

Following the analysis of differentially regulated cytokines and chemokines, we extended our investigation to explore the expression of genes involved in neuronal cell death, as they are associated with movement disorders. To assess these changes, we calculated the Z-score, representing the normalized gene expression for pathways relevant to these processes in both the MPTP compared to PBS and MPTP versus C-EV-treated MPTP groups. For the neuronal cell death pathway, we identified a Z-score of 1.232 (p = 8.65E-08) in the MPTP group compared to the PBS group, indicating activation of this pathway, while 0.665 (p = 2.03E-03) in the C-EV treated group compared to MPTP-intoxicated comparators. These data demonstrated deactivation following C-EV treatment (Panel 8A, Figure 8B). In the analysis, 13 genes were assessed in both the neurodegenerative and neuroprotective pathways. Among the top 10 hits, six genes [*LCN2, SERPINA3, PLA2G3, CDK1, SGK1,* and* PYCARD* (Apoptosis-associated Speck-like protein containing a CARD)] showed a trend of upregulation by MPTP compared to the PBS group, with *PLA2G3, SGK1,* and* OSMR* showing significant upregulation (p < 0.05). These genes are well documented for their roles in promoting cell death, consistent with MPTP neurodegeneration. For instance, neuronal cell death is mediated by these genes, including *LCN2*, which promotes neuroinflammation and neuronal death via oxidative stress and TNF-α 67, *SERPINA3* by NF-κB and RYR2 signaling 86, *PLA2G3* by glutamate-induced necrosis and calcium influx 87, CDK1 drives apoptosis via *FOXO1* (Forkhead box protein O1) phosphorylation and mitochondrial fission 88, *and SGK1* by activating the pro-apoptotic FOXO3a 89. *PYCARD*, an inflammasome adaptor, amplifies caspase-1-dependent cytokine release 90. The neuroprotective genes, including *OSMR*, *TRPV4, CDKN1A*, and *NAIP1* (NLR Family Apoptosis Inhibitory Protein 1), were also upregulated in MPTP mice (Figure 8B), indicating compensatory mechanisms. These genes confer neuroprotection by various mechanisms (s), such as *OSMR* by activating the JAK2/STAT3 pro-survival signaling pathway 91, *and TRPV4* enhances microglia migration to sites of damage 92. *CDKN1A* has been reported to reduce the production of proinflammatory cytokines in MPTP mice and simultaneously upregulate nuclear factor erythroid-related factor 2 (Nrf2), a regulator of antioxidative and anti-inflammatory responses 93. *NAIP1* protects dopaminergic neurons via PI3K/Akt signaling 94. Although these genes showed a trend of downregulation in C-EV-treated MPTP mice compared to those treated with MPTP alone, the differences were not significant (Figure 8C). These findings indicate that C-EV treatment may promote neuronal survival by attenuating pro-death pathways. We observed a Z-score of -0.396 (p = 4.99E-05) in the MPTP group compared to the PBS group. These data were assessed against known movement disorder pathways, while 0.029 (p = 3.10E-08) in the C-EV-treated MPTP mice compared to that of MPTP alone (Panel 9A, Figure 9B). Among the 30 genes associated with this pathway, the top-10 hits are shown in Figure 8D. Among the top 10 hit genes, five genes [*LCN2, SCN10A* (Sodium Voltage-Gated Channel Alpha Subunit 10), *ITGAX, TUBB6* (tubulin beta 6 class V), *and AQP1* (aquaporin 1)] showed a trend toward upregulation in the MPTP group compared to the PBS group. These include *LCN2*, a neuroinflammatory mediator 95; *SCN10A*, a NaV1.8 sodium channel linked to neuronal hyperexcitability 96; *ITGAX* encodes for CD11c, which is associated with α-synuclein transport in CD11c+ macrophages 97; and *TUBB6*, which encodes class V β-tubulin, whose higher expression contributes to muscle dystrophy 98. Additionally, *TUBB6* directly interacts with the brain LRRK2 kinase involved in PD 99 and AQP1, a marker of reactive astrocytes that modulates cortical α-synuclein pathology in advanced PD 100. All these genes showed a trend toward recovery in the C-EV-treated MPTP group compared to that in the MPTP group. Moreover, we observed a trend of downregulation in five neuroprotective genes [*dopamine β-hydroxylase (DBH*), *thyrotropin-releasing hormone (TRH*), nerve growth factor receptor), *GBX1* (gastrulation brain homeobox 1), and *CYP19A1* (Cytochrome P450 Family 19 Subfamily A Member 1)] in MPTP mice compared to the PBS group (Fig. 8D), however, no significant differences were observed in the expression levels of these genes in the C-EV-treated MPTP mice compared to those in the MPTP alone group. The functions of these genes include *DBH*, which restores norepinephrine synthesis 101; *TRH* analogs, which inhibit oxidative stress and asparagine endopeptidase (AEP)-mediated protein fragmentation 102; *NGFR*, which enhances neurogenesis 102; *GBX1*, which is critical for motor neuron survival and differentiation 103; *and CYP19A1*, which increases in the brain after neurotoxic damage and exerts neuroprotection through the synthesis of aromatase and estrogens 104. Additional genes associated with both neuronal cell death and movement disorder pathways are listed in Figure S3E. These genes play crucial roles in maintaining motor function, reinforcing the potential of C-EV treatment to restore motor pathways affected by MPTP. Together, these data indicate that C-EV treatment has probable neuroprotective and therapeutic effects. There was also a trend toward recovery in gene expression in the C-EV-treated MPTP mice relative to the MPTP group. Several genes, *CDKN1A, PLA2G3*, and *ITGAX*, remained significantly upregulated in C-EV-treated MPTP mice relative to the PBS control group.

### Inflammation-associated regulatory pathways

Next, we investigated the critical factors that regulate the production of pro-inflammatory and anti-inflammatory cytokines, focusing on the protein receptors, intermediate signaling proteins, and transcription factors (TFs) involved in these pathways. This analysis considered the top ten TF genes, comprising inflammatory and anti-inflammatory TFs. Among the five TF genes [*STAT3* (signal transducer and of transcription 3) 105, *NFAT5* (Nuclear Factor of Activated T cells 5) 106, 107, *SP1* (Specificity Protein 1) 108, *NF-κB* 109, and *HIF-1α* (hypoxia-inducible factor) 110 that regulate inflammatory responses, four showed a trend of upregulation in the MPTP group compared to the PBS group. We observed no changes in these genes in the C-EV-treated MPTP group compared to the MPTP alone group. No significant difference was observed between the PBS- and C-EV-treated MPTP mice (Figure 9A). Furthermore, we analyzed five TFs involved in anti-inflammatory responses: *FOXO1* 111, *CEBPA* (CCAAT/enhancer-binding protein alpha) 112, *SMAD3* (suppressor of mothers against apentaplegic 3) 113, *CREB1* (cAMP Response Element-Binding Protein 1), and Phosphatase and Tensin Homolog (*PTEN*). Among the five anti-inflammatory factors, *FOXO1, CEBPA*, and* SMAD3* showed a trend toward upregulation in the MPTP group compared to the PBS group. No further changes were observed in C-EV-treated MPTP mice compared to those observed with MPTP administration alone. Furthermore, the other two anti-inflammatory factors, *PTEN* 114 and *CREB1* 115, showed no change among the groups. However, no significant difference was observed between the C-EV-treated MPTP group and the PBS group (Figure 9A). Given that transcription factors directly regulate gene transcription but are themselves activated by upstream signaling proteins, we extended our analysis to investigate the receptors and intermediary signaling proteins critical for the activation of these transcription factors. Specifically, we analyzed 16 genes encoding receptors and signaling proteins involved in the activation of these pathways 116-124. Among the top 10 hits, eight genes, namely, *TCF7L2* (transcription factor 7 like 2), *TET2* (Ten-Eleven Translocation 2), *TICAM1* (TIR domain-containing adapter molecule 1), *TLR2* (toll-like receptor2), *CALCA* (Calcitonin Related Polypeptide Alpha), *TLR9* (Toll-like receptor 9), *MYD88* (*myeloid differentiation primary response 88*)*,* and *PARP1* [poly (ADP-ribose) polymerase-1], showed upregulation in the MPTP group compared to the PBS group. *TET2, TLR2, CALCA,* and* PARP1* showed a trend of downregulation in the MPTP + C-EV group compared to that in the MPTP alone group. *STING1* (stimulator of interferon genes 1) and *PPIF* (peptidyl-prolyl cis-trans isomerase F) levels showed no changes among the different groups. Additional genes associated with receptors and signaling proteins are listed in Figure S3F. However, no significant difference was observed in gene expression between the C-EV-treated MPTP and PBS groups (Figure 9B).

### Treg frequency

**Figure 10:**
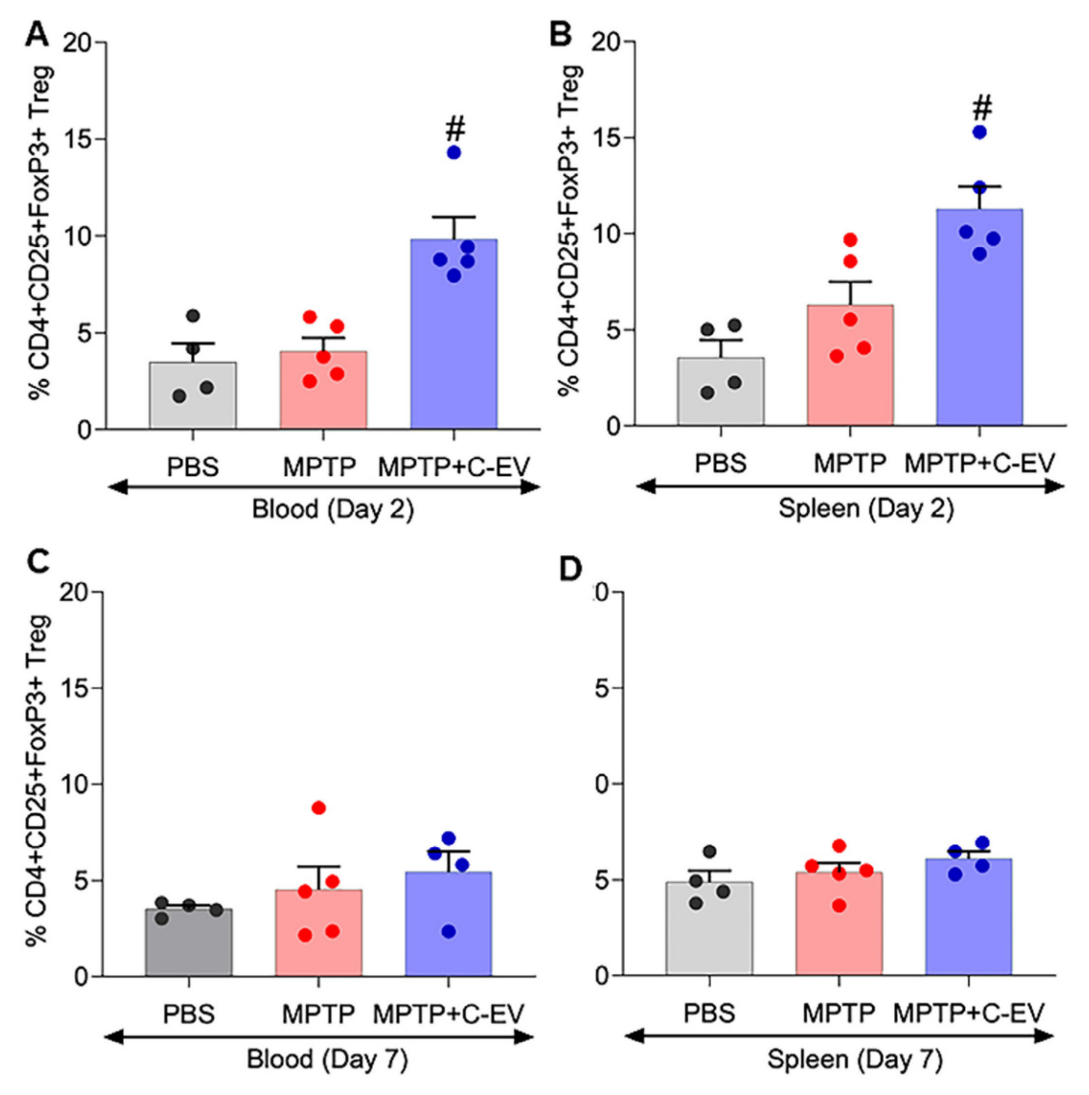
** Treg numbers in colostrum EV-treated MPTP mice.** (A) Quantification of CD4+CD25+FOXP3+ Treg cells in mouse whole blood after two days of MPTP intoxication. (B) Quantification of CD4+CD25+FOXP3+ Treg cells in the mouse spleen after two days of MPTP intoxication. (C) Quantification of CD4+CD25+FOXP3+ Treg cells in mouse whole blood after seven days of MPTP intoxication. (D) Quantification of CD4+CD25+FOXP3+ Treg cells in the mouse spleen 7 days after MPTP intoxication. N = 4 in PBS, N = 5 in MPTP, N = 5 in C-EV treated MPTP mice, N = 3 mice/group. Data are expressed as mean ± SEM. #p≤0.05 MPTP. Abbreviations: Treg, Regulatory T-cells; EVs, colostrum-derived extracellular vesicles.

Tregs have immunosuppressive functions, notably inhibiting T-effector cell-mediated neuroinflammation 125. Flow cytometry was performed to assess whether C-EV treatment influenced Treg frequency. For flow cytometric analysis of Treg frequency, gating was performed for CD3, CD4, CD8, CD25, and Foxp3 markers. The gating strategy for the different T cell subsets is shown in Figure S2A. Flow cytometric analysis showed that Treg frequency on day 2 (Figure 10A-B) following MPTP intoxication was significantly higher (p < 0.05) in the MPTP+C-EV group, both in the blood and spleen, compared to PBS or MPTP alone, implying that the C-EV treatment plays a significant role in enhancing the anti-inflammatory response by impacting Treg proliferation. In contrast, no significant change in Treg frequency was observed on day 7 post-MPTP administration, suggesting that the Treg response had fully subsided by day 7 of the experiment (Figure 10C-D). The CFSE assay was performed using splenic Tregs on day 7 post-MPTP administration to assess Treg function. However, no significant intergroup differences in Treg function were observed among the groups on day 7 (Figure S2B). These data imply that C-EV treatment plays a substantial role in Treg proliferation and consequently, the anti-inflammatory activities of the cells. This explains the signature role of Tregs. Although these effects were limited to anti-inflammatory effects, they nonetheless affected neuroprotective responses.

### Enrichment of miRNAs in the C-EVs

**Figure 11:**
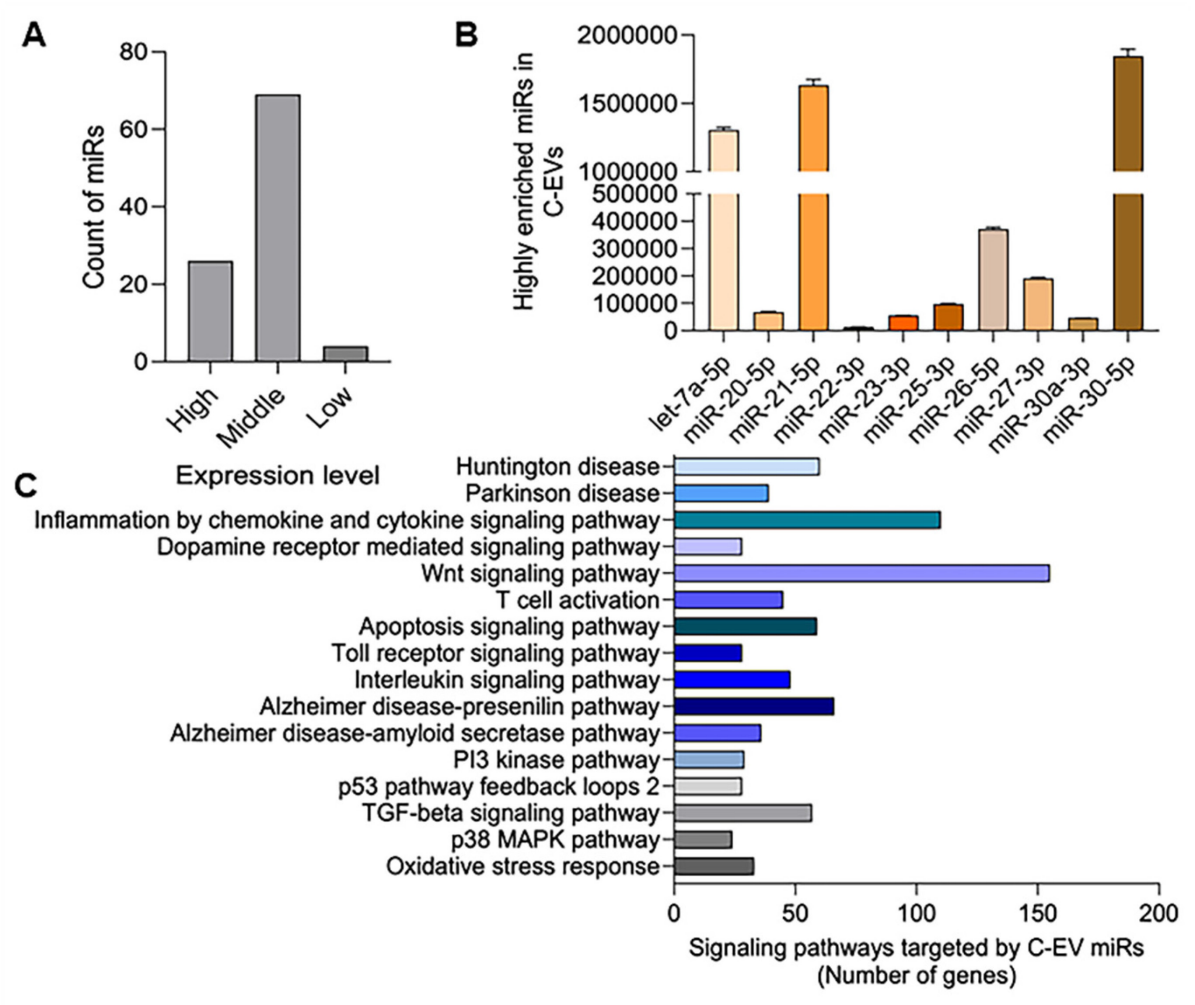
** Differentially expressed miRNAs in C-EVs.** (A) miRNA counts in C-EVs. (B) Differential enrichment of miRNAs in C-EVs. (C) Enrichment of pathways associated with miRNAs. N = 3.

C-EVs are enriched in miRNAs that can modulate recipient cell functions through their miRNA cargo. We performed small RNA sequencing on these C-EVs to elucidate the potential molecular mechanisms underlying the anti-inflammatory and neuroprotective effects of C-EVs in PD mice. Our analysis revealed 99 miRNAs in colostrum EVs, with 26 highly abundant miRNAs (≥20,000 reads) (Figure 11A). These 26 miRNAs belonged to 10 conserved miRNA families (Figure 11B), including miR-20-5p and miR-23 b-3p, which have been implicated in anti-inflammatory and neuroprotective processes in neurodegenerative diseases 126, 127. The potential functions and pathways of the genes targeted by C-EV-derived miRNAs were investigated using the PANTHER classification system, as shown in Figure 11C; some high-enrichment pathways were listed. Enrichment of miRNAs in C-EVs involves loading specific miRNAs, which can occur passively (by increasing cellular miRNA levels) or actively (using engineered methods, such as surface modification to create therapeutic agents or stable biomarkers for diagnosis), leveraging the ability of C-EVs to protect miRNAs from degradation. This process highlights the use of C-EVs as drug delivery systems, enhancing their activities over individual free miRNAs. This was used as an early event to better understand their abilities to affect cell biology. Large-scale, specific loading, and isolation remain issues for clinical translation.

### Loading of miRNAs

**Figure 12:**
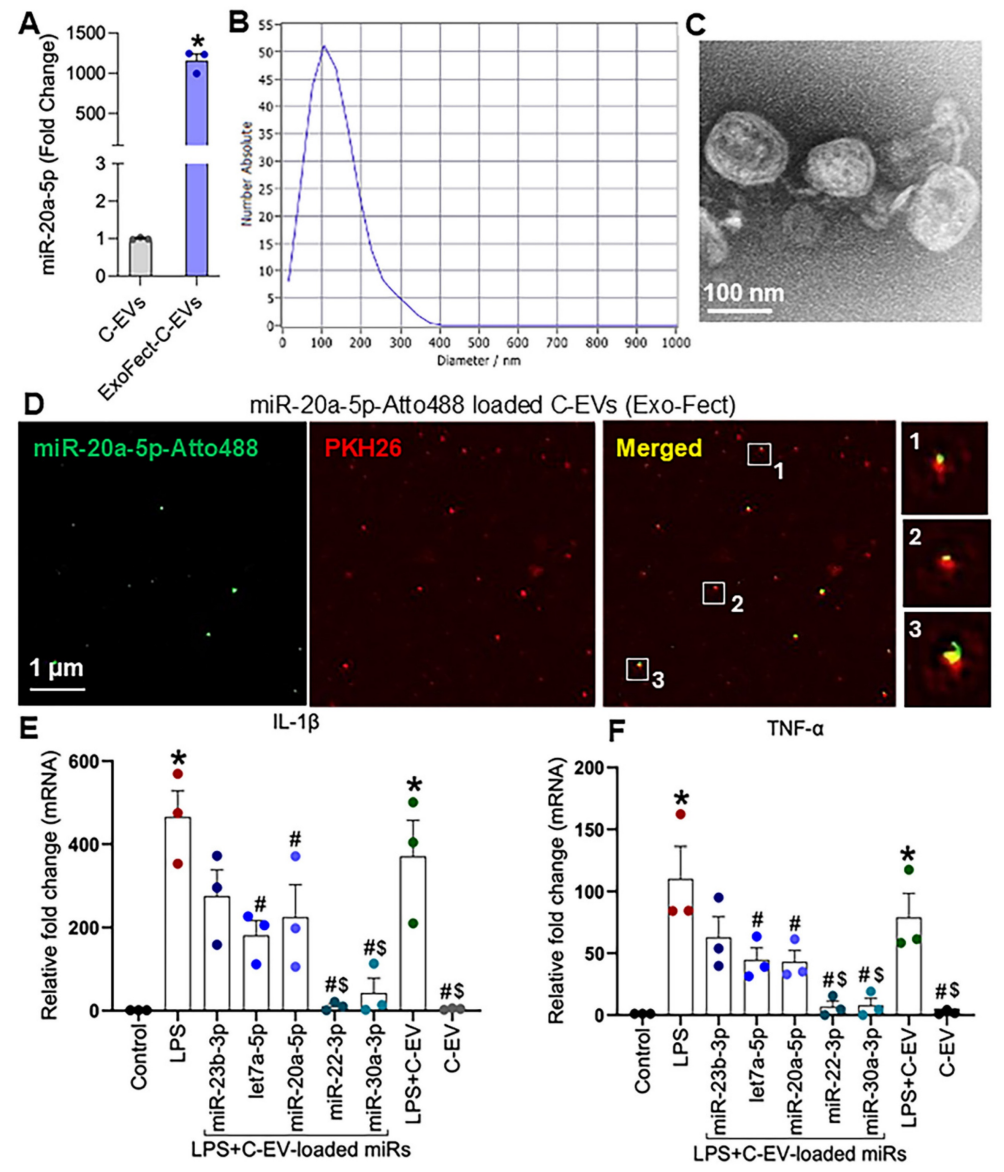
** Loading miRs into C-EVs.** (A) Real-time PCR quantification of miR-20a-5p loading efficiency in C-EVs following transfection with Exo-Fect. (B) Nanoparticle tracking analysis (NTA; NanoSight) of the size distribution of C-EVs after miR-20a-5p loading. (C) Transmission electron microscopy (TEM) image comparing the morphology of C-EVs post-transfection with miR-20a-5p using Exo-Fect. (D) Super-resolution microscopy of C-EVs loaded with miR-20a-5p-Atto488 (green) and membranes stained with PKH26 (red). Real-time PCR analysis of (E) IL-1β and (F) TNF-α mRNA expression in BV2 cells pretreated for 24h with either (i) naïve C-EVs or (ii) miR-loaded C-EVs, followed by LPS stimulation (2h). Data are expressed as mean ± SEM; N = 3/group. * p < 0.05 vs Ctrl; # p < 0.05 versus LPS; $ p < 0.05 versus C-EVs and LPS.

To determine whether miRs contribute to the anti-inflammatory effects of C-EVs, we loaded different miRs into C-EVs using the Exo-Fect transfection method. Quantitative PCR revealed that Exo-Fect enhanced miR-20a-5p levels in EVs by more than 1,000-fold (Figure 12A). Nanoparticle tracking analysis confirmed that loading of miRs in C-EVs did not alter the size distribution of C-EVs (Figure 12B). Consistent with this, TEM imaging showed no morphological changes in C-EVs after miR-20a-5p loading (Figure 12C), and super-resolution microscopy confirmed successful miRNA incorporation into EVs (Figure 12D). Similarly, additional miRs- miR-23b-3p, let7a-5p, miR-22-3p and miR-30a-3p were also loaded in the C-EVs. Next, we evaluated the functional impact of treating BV2 microglial cells with either naive C-EVs or miR-20a-5p, miR-23b-3p, let7a-5p, miR-22-3p, and miR-30a-3p-loaded C-EVs (24h pretreatment), followed by LPS stimulation (1 ng/mL, 2h). qPCR analysis demonstrated that all miR-loaded C-EV treatments significantly attenuated LPS-induced IL-1β and TNF-α expression, with miR-22-3p and miR-30a-3p-loaded C-EVs exhibiting superior anti-inflammatory effects compared to unmodified C-EVs (Figures 12E, 12F). The LPS model of PD 128 has been widely studied to induce neuroinflammation and mimic key features of the disease, associated with activation of microglia and release of inflammatory molecules, leading to the degeneration of neurons in the substantia nigra.

## Discussion

Parkinson's disease (PD) is the second most common neurodegenerative disease after Alzheimer's disease (AD) and the most prevalent neurodegenerative movement disorder. Motor function deficits include tremors, bradykinesia, rigidity, and postural instability, all of which result from reduced dopamine neurotransmitter levels due to the loss of dopaminergic neurons. Current PD treatments are palliative and do not affect dopaminergic loss or disease progression. Additionally, although current therapeutics, such as levodopa, dopamine receptor agonists, and MAO-B inhibitors, provide greater availability of dopamine to increase motor control, they do not address the underlying cause of the disease or neuronal loss. Moreover, long-term drug use can lead to adverse reactions 129, 130.

We investigated C-EVs as a natural therapy for PD based on their known anti-inflammatory and wound-healing properties 33, 131-133. This success in achieving a multifaceted approach to PD supports the association between inflammation and neuroprotection. The overarching idea is that colostrum EVs promote cell proliferation, migration, and intercellular tube formation and provide cargoes containing anti-inflammatory and immune-modulating factors. This facilitates the transition from inflammation to cell and tissue repair processes. 33, 131, 132. Thus, colostrum is a promising anti-inflammatory and neuroprotective agent. While no cure currently exists for PD, new modalities that attenuate neuroinflammation may also mitigate neurodegeneration. One is C-EVs which interfere with disease and facilitate restoration of brain health.

The current study demonstrated that C-EVs exhibit potent anti-inflammatory and neuroprotective effects in a neuroinflammation and dopaminergic (DAergic) neurodegeneration animal model. Two days after MPTP intoxication, the number of activated microglia increased 4-fold, and by day seven, 84% of the nigral DAergic neurons were lost. However, C-EV treatment reduced the number of activated microglia by 43% and rescued 33% of nigral DAergic neurons. Interestingly, C-EV treatment had no significant neuroprotective effect on dopaminergic terminals in the striatum, probably because of variations in the brain region-specific distribution of C-EVs. Several studies by our 134-137 group and others 134-137 have demonstrated a loss of DA neurons that can be protected by pharmaceuticals in MPTP mice. However, the protective effects of C-EVs against neuronal death have not been well studied. Our study demonstrated, for the first time, that C-EVs have neuroprotective properties in a PD model. Furthermore, DA neuronal loss is supported by transcriptome-based changes in genes associated with the pathways of neuronal death. Transcriptomic analysis of nigrostriatal tissues indicated that MPTP upregulates several genes related to neuronal cell death and movement disorders. This includes lipocalin (*LCN2*), which mediates neuronal cell death by activating nitrosative and oxidative stress as well as inflammation 138, *SERPINA3* which can potentiate neuroinflammation 86, *PLA2G3* which potentiates oxidative stress 87; *CDK1*, which can potentiate cell death by phosphorylating the FOXO1 transcription factor 88; *SGK1*, which is involved in dopaminergic neuronal loss in PD 139; *PYCARD* 140, *OSMR* 141-143, which have been implicated in neuroinflammatory responses leading to neuronal cell death; and *TRPV4*, a calcium-permeable cation channel that mediates ER stress and activation of inflammation, and has been reported to be involved in the loss of DA neurons in PD mice 143. *CDKN1A*, which regulates the cell cycle, was also upregulated. DNA damage 144 often leads to cell death 145. However, treatment with C-EVs reduced the expression of these genes. Interestingly, treatment of MPTP mice with C-EVs increased the expression of neuroprotective genes, such as *NGB, NGFR, NAIP1-NLR* family, apoptosis inhibitory protein 1, and neuron-derived orphan receptor 1 (*NR4A3*). Prior studies have also demonstrated alterations in *LCN2* 95, 146, *SGK2* 139, *OSMR* 147, and *TRPV4* 143 in different PD models, this is the first report to demonstrate the neuroprotective role of C-EVs in genes associated with neuronal death pathways. As PD is associated with motor dysfunction, we assessed the gene alterations related to movement disorders. Our transcriptomic analysis demonstrated that genes associated with the enhancement of movement disorders, such as *LCN2, SCN10A, ITGAX, TUBB6, AQP1, CD44, MUSK, APOD, LGALS3, FOXA2, LRG1, GPD1,* and* HSPA1A*, were upregulated in the MPTP group, and that C-EV treatment decreased the expression of these genes, thus demonstrating the protective role of C-EVs in movement disorders. Genes associated with neuroprotection and dopamine uptake were upregulated in the C-EV-treated MPTP group. Few were altered in PD models by others, including *LCN2* 95, 146, *SCN10A* 148, *CD44* 78, *APOD* 149, *FOXA2* 78, *APOD* 149, *FOXA2* 150, *HSPA1A* 102, *GSTP1* 150, and *TRH* 102. Nonetheless, such detailed transcriptomic analysis has not been reported by C-EV treatment. After demonstrating the neuroprotective role of C-EVs, we found that neuroinflammation was associated with neurodegeneration. While increased levels of activated microglia after MPTP intoxication validate previous reports of cellular immune responses in afflicted brain regions 151-153, detailed process pathways have not yet been reported. Moreover, activation status may also reflect the differential microglial function between MPTP-intoxicated and C-EV-treated mice 154, 155. Total RNA sequencing and transcriptomic analysis of nigral and striatal tissues from mice with two days of MPTP intoxication demonstrated increased expression of genes associated with inflammatory responses, recruitment, and activation of macrophages and granulocytes. C-EV treatment of MPTP mice downregulated the expression of most of those genes. We also showed that MPTP upregulated the genes associated with a spectrum of pro-inflammatory microglial phenotypes 156.

Transcriptomic analysis of the midbrain and striatal tissues also revealed that MPTP increased the NLR gene family expression associated with inflammasome activation. In contrast, C-EVs downregulated many of those inflammasome-associated genes. Similarly, western blot analysis further validated that MPTP induced upregulation of NLRP1, 3, 6, and 10 in the mid-brain and striatal tissues. C-EV treatment of MPTP mice significantly reduced levels of those NLRP proteins. Moreover, downstream inflammasome effector proteins such as caspase 1, IL-18, and IL-1β were upregulated with MPTP treatment and downregulated to background levels with C-EV-treatment. Overall, these findings validate the regulatory effects of C-EVs on inflammatory responses at the transcript and protein levels. Additionally, our RNA-seq data demonstrated the co-expression of classical “M1-like” genes (*CD68, CD32, and CD86*) together with disease-associated microglial (DAM) or repair markers, such as TREM2 and APOE 157, 158. CD74, which is upregulated in the MPTP+C-EV condition, is increasingly recognized as a marker of activated, immunologically engaged microglia regulated by TGF-β signaling rather than a strict indicator of pro-inflammatory toxicity 159. Additionally, MPTP intoxication results in a distinct microglial activation signature characterized by the upregulation of *CD68, CD16, CD32, and CD86*, indicating microglial priming for immune activation 160. Elevated Fc receptors (*CD16, CD32*) 161 and the cell activation marker CD64 reflect increased phagocytic activity 162, whereas CD86 suggests an increased co-stimulatory potential for T-cell activation 163. This transcriptional profile represents a primed microglial phenotype, providing a signal for inflammasome activation by increasing NF-kB-dependent transcription of NLRPs and pro-IL-1β 164. Consistent with this primed state, the inflammasome panel in Figure 6 shows the upregulation of the inflammasome pathway in the MPTP model. Together, these data indicate that microglia transition from a primed to an activated state, driving the execution phase of neuroinflammation 164. C-EV treatment effectively modulated this response. At the transcriptional level, C-EVs dampen the expression of *CD68, CD16, CD32,* and *CD86*, indicating lower phagocyte activation. CD74 and H2-Eb expression showed a slight increase after C-EV treatment. Both are invariant chains of MHC II and play important roles in pathogen loading 165. Although they classically signify M1 phenotype activation, they also help protect against injury and promote repair 166, 167. Here, we speculate that C-EVs act as a moderator, not switching the microglial polarity but maintaining a balanced condition and preventing the secretion of proinflammatory cytokines. At the protein level, C-EVs significantly suppressed NLRP3, NLRP1, NLRP6, and NLRP10, reduced caspase-1 cleavage, and decreased the expression levels of mature IL-1β and IL-18. This demonstrates that C-EVs suppress immune vigilance from inflammasome hyperactivation, redirecting microglia toward a more regulatory phenotype that no longer propagates a proinflammatory response. Together, these data indicate that C-EVs dampen inflammasome signaling and the IL-1β/IL-18 axis, while some antigen-presentation/interferon-related genes (e.g., CD74) remain elevated, findings that are fully compatible with modern concepts of microglial diversity and disease-associated states 157, 158.

Proinflammatory cytokines and chemokines are upregulated after MPTP intoxication and have been recorded in other PD models 168, 169. Indeed, protein array analysis of tissues from the nigrostriatal axis revealed that MPTP upregulated several proinflammatory cytokines and chemokines, including IL-1β, IL-12, IL-17, IFN-γ, CXCL10, CCL9, and CCL20. Simultaneously, treatment of these mice with C-EVs downregulated proinflammatory mediators. Similarly, receptors and binding proteins associated with inflammation showed a trend of upregulation in the MPTP group, but were downregulated with C-EV treatment. Overall, C-EVs played a potent suppressive role in most inflammatory components in this model.

Finally, we sought to determine the cellular mechanism by which C-EVs attenuate proinflammation and mitigate DAergic neurodegeneration in this model. We have acquired substantial evidence for the anti-inflammatory and neuroprotective effects of Tregs in PD models and their association with improved clinical scores in clinical trials of PD 10, 170-173. For these studies, we showed that in C-EV-treated MPTP mice, Treg frequencies in blood and spleens were significantly increased by day two after MPTP intoxication; however, by day seven, Treg frequencies in the peripheral immune compartments and function of splenic Tregs from mice treated with or without C-EVs had returned to background levels. These data support the notion that C-EVs induce Tregs control neuroinflammation and DAergic neurodegeneration in the MPTP PD model. Additionally, we observed an increase in Treg frequency on day two, which was normalized by day seven, mostly due to the adoptive Treg transfer or *in vivo* Treg expansion not being linked to optimal timing and number of Tregs. Key mechanisms are linked to the release of anti-inflammatory cytokines and suppression of pro-inflammatory immune cells, although the specific role, timing of Treg, and their infiltration into the brain are complex and vary depending on factors that are disease-linked and secretory profile mechanisms 170, 174. MicroRNAs (miRNAs), circular RNAs (circRNAs), Y-RNAs, long noncoding RNAs (lncRNAs), small nuclear RNAs (snRNAs), small nucleolar RNAs (snoRNAs), transfer RNAs (tRNAs), and piwi-interacting RNAs (piRNAs) are potential cargoes contained within C-EVs. While speculative as a therapy each RNA family have been demonstrated to affect cell and tissue homeostasis and regenerative C-EVs properties. To further delve deeper into the C-EV cargoes, our small RNA-seq results showed the presence of 10-top hit anti-inflammatory and neuroprotective miRNAs, which contribute to the neuroprotective role of colostrum EVs in the MPTP mouse model. Furthermore, our study revealed that miR-23b-3p, let-7a-5p, miR-22-3p, and miR-30a-3p, which are enriched in these C-EVs, exhibit strong anti-inflammatory properties, with let-7a-5p, miR-22-3p, and miR-30a-3p being the most potent among them. Future studies should include additional anti-inflammatory miRNAs enriched in C-EVs and their functions in animal models of PD. Therefore, in the present study, we concluded that C-EVs regulate neuroinflammation and elicit neuroprotection of dopaminergic neurons in Parkinsonian-type neurodegeneration. We demonstrated that C-EVs induce anti-inflammatory and neuroprotective activities. However, future studies are needed to determine the cellular and molecular mechanisms by which C-EVs affect Treg function and sustain a stable brain microenvironment. Future detailed assessment of C-EV cargos and the resulting improvements in neuroprotective outcomes will provide insights into the benefits of colostrum on translational disease outcomes. These findings support the development of additional therapeutic strategies for combating neurodegenerative diseases. Future research will elucidate how C-EV loading with additional agents determines the effectiveness of this therapeutic approach in using immune and neuronal mediators to attenuate neuroinflammation, mitigate neurodegeneration, and repair brain damage invivo.

We also acknowledge the limitations of this study that could affect the clinical translation of C-EV. Colostrum composition can vary due to maternal factors such as age, parity, diet, and collection timing 175, which can affect the C-EV yield and cargo. To address this, properly standardized protocols are required for donor screening and C-EV isolation. Colostrum is mostly safe for neonates; however, adult recipients may exhibit immune responses to bioactive C-EVs 176, especially those from xenogeneic sources such as bovine colostrum, which necessitates immunotoxicology studies and dose optimization. The regulatory approval procedure is complex, as agencies such as the FDA and EMA develop evolving guidelines for C-EV-based therapeutics. Therefore, for clinical translation, C-EVs are derived from food-grade, non-immunogenic materials with a long history of human consumption. This simplifies their translational path compared to other EV sources, which often face scrutiny due to the potential risk of immunogenicity**.** Furthermore, our studies demonstrate that C-EVs from different batches exhibit similar functional efficiency; however, regulatory guidelines approved by the FDA will be followed prior to clinical use. In addition, C-EVs have several translational advantages. First, they are in terms of yield, scalability, and cost-effectiveness**.** Bovine colostrum is a rich, renewable, and low-cost source of C-EVs. It offers several translational advantages for large-scale production compared to EVs derived from cultured stem cells 37. *First* is quantity. *Second* is safety. EVs are derived from food-grade, non-immunogenic materials with a long history of human consumption. This greatly simplifies their regulatory path compared to stem cell-based products, which often face scrutiny due to the potential risks of oncogenesis and immunogenicity 131. *Third*, C-EVs have demonstrated excellent stability under gastrointestinal conditions and effective uptake across biological barriers, including the blood-brain barrier 177. *Fourth*, C-EVs modulate not only central nervous system inflammation but also systemic immune responses and gut-brain axis interactions, which are increasingly recognized as critical in PD pathogenesis 178. *Fifth*, we functionally validated anti-inflammatory miRs and showed enhanced cytokine suppression with miR-loaded C-EVs. These results highlight the therapeutic potential of colostrum EVs as drug-like carriers for future treatments 37. *Sixth*, a food-derived starting material with an established safety history may face fewer perceptions and supply chain hurdles than human cell-sourced products, facilitating early clinical exploration. Another limitation of this study is that brain region-specific neuroprotection was not provided by C-EVs against MPTP-induced striatal degeneration in the study. The latter was due, in large part, to the MPTP itself. Nonetheless, significant neuroprotection was observed in the substantia nigra, which was linked to the brain subregion-specific distribution of C-EVs. Additionally, although gene expression patterns were uniformly statistically significant across the groups, z-scores denoting activation pathway patterns were significantly altered, demonstrating activation in the recorded inflammatory and neurodegenerative pathways by the MPTP group, while activation of these pathways were attenuated by C-EV treatments. Additionally, qPCR analysis showed that the pro-inflammatory CCR6, S100a4, CD68, CD32, Msr1, and Arg1 genes, which were upregulated in the MPTP group, were significantly reduced after C-EV treatment in MPTP mice.

Other sources of EVs, such as mesenchymal stem cell-derived EVs (MSC-EVs), have been shown to cross the blood-brain barrier, attenuate neuroinflammation, and rescue dopaminergic neurons in an induced PD mouse model 179. Mechanistically, MSC-EVs mediate these effects by transferring neuroprotective molecules such as miRNAs and anti-inflammatory proteins 179. Similarly, in a rotenone-induced rat Model of PD, neural-induced human adipose stem cell-derived exosomes (NI-hADSC-Exo) significantly reduced neurodegeneration, leading to improved motor function and increased survival of dopaminergic neurons by lowering levels of pathogenic oligomeric phosphorylated α-synuclein (p-S129 α-syn) and suppressing astrocytic and microglial activation 180. Similarly, our study demonstrated that C-EVs exerted robust anti-inflammatory and neuroprotective effects in an MPTP-induced mouse model of PD. Specifically, C-EV treatment reduced the number of activated CD11b⁺ microglia and preserved TH⁺ neurons in the substantia nigra of the brain. Unbiased transcriptomic profiling and targeted protein validation revealed the suppression of inflammasome signaling pathways (reduced expression of NLRP1, NLRP3, and NLRP6, as well as caspase-1) and downstream proinflammatory cytokines (IL-1β and IL-18). These *in vivo* findings are consistent with our *in vitro* results, which showed that C-EV-loaded miR-enriched preparations suppressed LPS-induced IL-1β and TNF-α expression in BV2 microglial cells. Consistent with our findings, these miRNAs have been shown to be neuroprotective in multiple neurodegenerative diseases. This includes miR-23b-3p 177 and let7a-5p 181 in AD, miR-20a-5p in PD 182, miR-22-3p in cerebral ischemia 183, AD 184, and PD 185. Additionally, compared to MSC-EVs and synthetic nanoparticles, C-EVs offer several distinct translational advantages in preclinical PD models. MSC-EVs have shown neuroprotective effects via the delivery of BDNF and miRNAs in 6-OHDA and rotenone models 43, 186. Likewise, synthetic nanoparticles can be engineered for brain targeting 187-189 and have been demonstrated to deliver L-DOPA 190 and antioxidants 191. However, both approaches face key limitations. Synthetic nanoparticles often require chemical modification for biocompatibility and brain access, yet still pose risks of immunogenicity and poor CNS biodistribution 192-194. Although biologically potent, MSC-EVs suffer from batch variability and scalability issues associated with the donor and culture conditions 195. In contrast, C-EVs are naturally enriched with anti-inflammatory and neuroprotective molecules 131, 196, show excellent safety from a food-grade, non-immunogenic source, and can be delivered orally or intranasally with stability across biological barriers 177, making them a highly scalable and clinically promising alternative to pharmaceutical drugs.

## Conclusions

C-EVs demonstrate anti-inflammatory activities by modulating the recruitment and activation of cellular immunity, transforming microglial phenotypes, activating TLR-genes, four canonical inflammasome complexes, and reducing pro-inflammatory cytokines, chemokines, and neurotoxic pathways through the regulation of *NF-κB, STAT3, HIF1A, FOXO1, SMAD3, CREB1,* and* PTEN*. The demonstrated anti-inflammatory responses lead to neuroprotection in the brain. This was demonstrated by C-EV-mediated rescue of TH+ dopaminergic neurons in the SN. These neuroprotective effects seem to be brain region-specific, as no changes in the loss of striatal termini were observable in this model. Colostrum contains numerous bioactive compounds that serve as nutrients and contain high levels of antioxidants and anti-inflammatory factors, including tocopherols, vitamins, biliverdin, pentasaccharides, gangliosides, and probiotics 197. Additionally, previous studies have demonstrated that EVs derived from different body fluids, including saliva, cerebrospinal fluid (CSF), amniotic fluid, and breast milk, have therapeutic or diagnostic potential 198, 199. This study for the first time demonstrated that bovine-colostrum-derived EVs have both brains targeting and disease attenuating potential. C-EVs demonstrate anti-inflammatory and neuroprotective activities. Taken together, C-EVs may represent a novel therapy for PD. Additionally, this study demonstrates that C-EVs could be beneficial beyond PD, potentially for AD, cerebral ischemia, stroke, multiple sclerosis, and amyotrophic lateral sclerosis, due to the enrichment of neuroprotective miRNA cargoes, as well as a minimal immunogenic response and high yield. Therefore, the results of the present study offer potential translational opportunities.

## Supplementary Material

Supplementary methods and figures.

## Abbreviations

BCL2A1d: B cell leukemia/lymphoma 2 related protein a1d
CCL21: C-C motif chemokine ligand 21
CCR2: C-c motif chemokine receptor 2
CSF: Cerebrospinal fluid
H2-Eb1: MHC class ii, e beta
LCN2: Lipocalin 2
SLC6A4: Solute carrier family 6 member 4
AD: Alzheimer's disease
AEP: Asparagine endopeptidase
Alix: ALG-2 interacting protein X
AQP1: Aquaporin 1
Arg1: Arginase 1
BAX: Bcl-2-associated X
BCL2A1a: B cell leukemia/lymphoma 2 related protein A1a
CALCA: Calcitonin related polypeptide alpha
CAPG: Capping actin protein, gelsolin-like
CCL19: C-C motif chemokine ligand 19
CCL20: C-C motif chemokine ligand 20
CCL4: C-C motif chemokine ligand 4
CCP: Cellular component pathways
CCR6: C-C motif chemokine receptor 6
CD163: Cluster of Differentiation 163
CD206: Cluster of Differentiation 206
CD301: C-type lectin domain family 10, member a
CD33: CD33 molecule
CD44: CD44 molecule
CD63: Cluster of differentiation 63
CD64: Cluster of Differentiation 64
CD74: Cluster of Differentiation 74
CD82: Cluster of differentiation 82
CD86: Cluster of differentiation 86
CDK: Cyclin-dependent kinase
CDKN1A: Cyclin dependent kinase inhibitor 1a
CEBPA: CCAAT/enhancer-binding protein alpha
CFSE: Carbosyfluorescein succinidyl ester
Chil3: Chitinase-like 3
circRNAs: Circular RNAs
c-JUN: Transcription factor jun
CNS: Central nervous system
CREB1: Camp Response Element-Binding Protein 1
CSF2RB: Colony stimulating factor 2 receptor subunit beta
CT: Computed tomography
CYP19A1: Cytochrome p450 family 19 subfamily a member 1
Cyt c: Cytochrome C
DAB: Diaminobenzidine
DAergic: Dopaminergic
DAT: Dopamine transporter
DBH: Dopamine β-hydroxylase
DEG: Differential gene expression
DLS: Dynamic light scattering
EAE: Experimental autoimmune encephalomyelitis
EDTA: Ethylenediaminetetraacetic acid
ESR1: Estrogen receptor 1
EVs: Extracellular vesicles
FDR: False discovery rate
FOXO1: Forkhead box protein O1
GBX1: Gastrulation brain homeobox 1
GGT5: Gamma-glutamyltransferase 5
GM-CSF: Granulocyte-macrophage colony-stimulating factor
HIF-1α: Hypoxia inducible factor 1 alpha
IL-10: Interleukin-10
IL12RB1: Interleukin 12 receptor subunit beta 1
IL1r1: Interleukin 1 receptor, type 1
IL-2: Interleukin-2
IL4R: Interleukin 4 receptor
ITGAX: Integrin subunit alpha X
LAMP1: Lysosomal-associated membrane protein 1
LCN2: Lipocalin
LGALS3: Galectin 3
lncRNAs: Long noncoding RNAs
LPA: Lysophosphatidic acid
MAO-B: Monoamine oxidase B
MCL-1: Myeloid cell leukemia-1
miRNAs: Micro RNAs
MPTP: Methyl-4-phenyl-1,2,3,6-tetrahydropyridine
MPTP-HCL: MPTP hydrochloride
Mrc1: Mannose receptor, c type 1
MSC-EVs: Mesenchymal stem cell-derived EVs
Msr1: Macrophage scavenger receptor 1
MYD88: Myeloid differentiation primary response 88
NAIP1: Nlr family apoptosis inhibitory protein 1
NFAT5: Nuclear Factor of Activated T cells 5
NF-Ƙb: Nuclear Factor-κb
NGFR: Nerve growth factor receptor
NO: Nitric oxide
NR4A3: Neuron-derived orphan receptor 1
Nrf2: Nuclear factor erythroid 2-related factor 2
OCT3: Organic cation transporter
OSMR: Oncostatin m receptor
PARP1: Poly (ADP-ribose) polymerase-1
PBS: Phosphate buffer saline
PC1-PC5: Principal components 1-5
PDI: Polydispersity index
PFA: Paraformaldehyde
PILRB2: Paired immunoglobin-like type 2 receptor beta 2
piRNAs: Piwi-interacting RNAs
PLA2G3: Phospholipase A2 group III
PPAR-γ: Peroxisome proliferator-activated receptor gamma
PPIF: Peptidyl-prolyl cis-trans isomerase F
PRKCD: Protein kinase C delta
PTEN: Phosphatase and Tensin Homolog
PVDF: Polyvinylidene fluoride
PYCARD: Apoptosis-associated Speck-like protein containing a CARD
RNA-seq: RNA sequencing
ROS: Reactive oxygen species
S100A4: S100 calcium-binding protein A4
Saa3: Serum amyloid A 3
Scarb1: Scavenger receptor class b member 1
SCN10A: Sodium voltage-gated channel alpha subunit 10
SERPINA3: Serpin family A member 3
SGK1: Serum/glucocorticoid regulated kinase 1
SL6A4: Solute carrier family 6 member 4
SMAD3: Suppressor of mothers against apentaplegic 3
snoRNAs: Small nucleolar RNAs
SNpc: Substantia nigra pars compacta
snRNAs: Small nuclear RNAs
SOCS3: Suppressor of cytokine signaling 3
SP1: Specificity protein 1
Sphk1: Sphingosine kinase 1
Sphk2: Sphingosine kinase 2
STAT3: Signal transducer and activator of transcription 3
STING1: Stimulator of interferon genes 1
TAMs: Tumor-associated macrophages
TCF7L2: Transcription factor 7 like 2
TEM: Transmission electron microscopy
TET2: Ten-eleven translocation 2
TF: Transcription factors
TGM2: Transglutaminase 2
TH+: Tyrosine hydroxylase
TICAM1: TIR domain-containing adapter molecule 1
TLR2: Toll-like receptor2
TLR4: Toll-like Receptor 4
TLR9: Toll-like receptor 9
TNF-α: Tumor necrosis factor-alpha
Treg: Regulatory T cells
TREM2: Triggering Receptor Expressed on Myeloid Cells 2
Tresp: Tresponder
TRH: Thyrotropin-releasing hormone
tRNAs: Transfer RNAs
TRPV4: Transient receptor potential cation channel subfamily v member 4
TSG101: Tumor susceptibility gene 101
TTBS: Tris-Buffered Saline with 1% Tween 20
TUBB6: Tubulin beta 6 class V
VMAT2: Vesicular monoamine transporter 2
VWF: Von Willebrand factor
