# Supplementary material for: Colostrum extracellular vesicles are neuroprotective in models of Parkinson's disease
Source: Theranostics. 2026 Jan 22;16(8):4190–223. doi: 10.7150/thno.128257 (PMC12905751; doi:10.7150/thno.128257)
Supplement: Supplementary file 1 — Supplementary methods and figures. [file thnov16p4190s1.pdf]

Reagents

The following antibodies and reagents were used in this study: anti-NLRP6 (cat. No. AB314498, Abcam, MA, USA), anti-NLRP3 (Cat. No. AB283819, Abcam, MA, USA), rabbit anti-NLRP1 (Cat. No. NBP1-54899; Novus Biologicals, CO, USA), anti-NLRP10 (Cat. No. MAB6606; R&D Systems, Minneapolis, MN, USA), anti-AIM2 (cat. No. 63660T; Cell Signaling Technology), anti-caspase-1 (Cat. No. 24232T; Cell Signaling Technology), anti-IL-18 (Cat. No. 57058S; Cell Signaling Technology, MA, USA), rabbit anti-IL-1β mAb (Cat. No. 31202T; Cell Signaling Technology, Danvers, MA, USA), and HRP-mouse anti-rabbit IgG (Cat. No. sc-2357, Santa Cruz Biotechnology, TX, USA), and horseradish peroxidase (HRP)-goat anti-mouse IgG (Cat. No. STN-271437, Abcam, MA, USA), anti-calnexin (Cat. No. ab22595, Abcam, MA, USA), anti-TSG101 (Cat. No. ab225877, Abcam, MA, USA), anti-CD63 (Cat. No. ab134045, Abcam, MA, USA), anti-CD81 (Cat. No. ab109201, Abcam, MA, USA), anti-LAMP1 (Cat. No. ab24170; Abcam), rabbit anti-rat antibodies (Cat. No. BA-4000; Vector Laboratories, CA, USA), anti-CD11b (5C6) (Cat. No. MCA711; Bio-Rad, CA, USA), casein antibody (Cat. No. ab166596, Abcam, MA, USA), PerCP-Cy5.5-anti-CD3 (Cat. no. 45-0037-42; eBioscience, CA, USA), PE-Cy7-anti-CD4 (cat. No. 25-0042-82; eBioscience, CA, USA), FITC-anti-CD8 (Cat. No. 11-0088-42; eBioscience, CA, USA), PE-anti-CD25 (Cat. No. 12-0251-82; eBioscience, CA, USA), anti-TH (Cat. No. 657012, CalBiochem, MA, USA), biotinylated goat anti-rabbit IgG (H+L) (Cat. No. BA-1000, Vector Laboratories, CA, USA) and a normal rabbit serum blocking solution (Cat. No. S-5000-20; Vector Laboratories, CA, USA) and normal goat serum blocking solution (Cat. No. S-1000-20; Vector Laboratories, CA, USA), ABC Elite PK-6100 Vectastain Elite Peroxidase (Cat. No. PK-6100; Vector Laboratories, CA, USA) and hydrogen peroxide solution (Cat. No. 7722-84-1, Sigma-Aldrich, MO, USA), and ABC Solution Biotin Avidin Peroxidase (Cat. No. PK4000; Vector Laboratories, CA, USA), and FACS Lysing Solution (Cat. No. BD 34902, eBioscience, CA, USA), Permeabilization Buffer (10X) (Cat. No. 00-8333-56, eBioscience, CA, USA), Fixation/Permeabilization Diluent (Cat. No. 00-5223-56, eBioscience, CA, USA), Fixation/Permeabilization Concentrate (Cat. No. 00-51-123-43, eBioscience, CA, USA) 30% Acrylamide/Bis Solution 37.5:1 (Cat. No. 1610158; Bio-Rad, CA, USA), polyvinylidene fluoride (PVDF) membranes (Cat. No. IPVH00010, Millipore Sigma, MO, USA). EasySep™ Mouse CD4+CD25+ regulatory T cell isolation kit II (StemCell Technologies).

Supplementary table and figures

Table 1: C-EV-characterization	Size (nm)	Particle Concentration (particles/mL)	RNA Concentration (µg/mL)	Protein Concentration (mg/mL)
Milk Collected 24 Hours After Birth	137±3	$1.83\times10^{11}\pm2.7\text{X}10^{5}$	42.7±3.7	16.17±1.5
Milk Collected 120 Hours After Birth	147±2	$8.36\times10^{10}\pm8.9\text{X}10^{4}$	25.1±2.8	5.71±2.1

Supplementary Table 1. Milk-derived EV characterization at different time points: A comparison between particles isolated from colostrum collected 24 h after birth and colostrum collected 120 h after birth. NTA was used to determine the particle size and concentration, and a Quant-it RiboGreen RNA Assay Kit was used to determine the RNA concentration. Protein concentration was determined using the bicinchoninic acid assay. Data are expressed as mean ± SEM. n=3/group. Abbreviations: NTA: nanoparticle tracking analysis; BCA: Bicistronic Acid; C-EVs: Colostrum Derived Extracellular Vesicles.

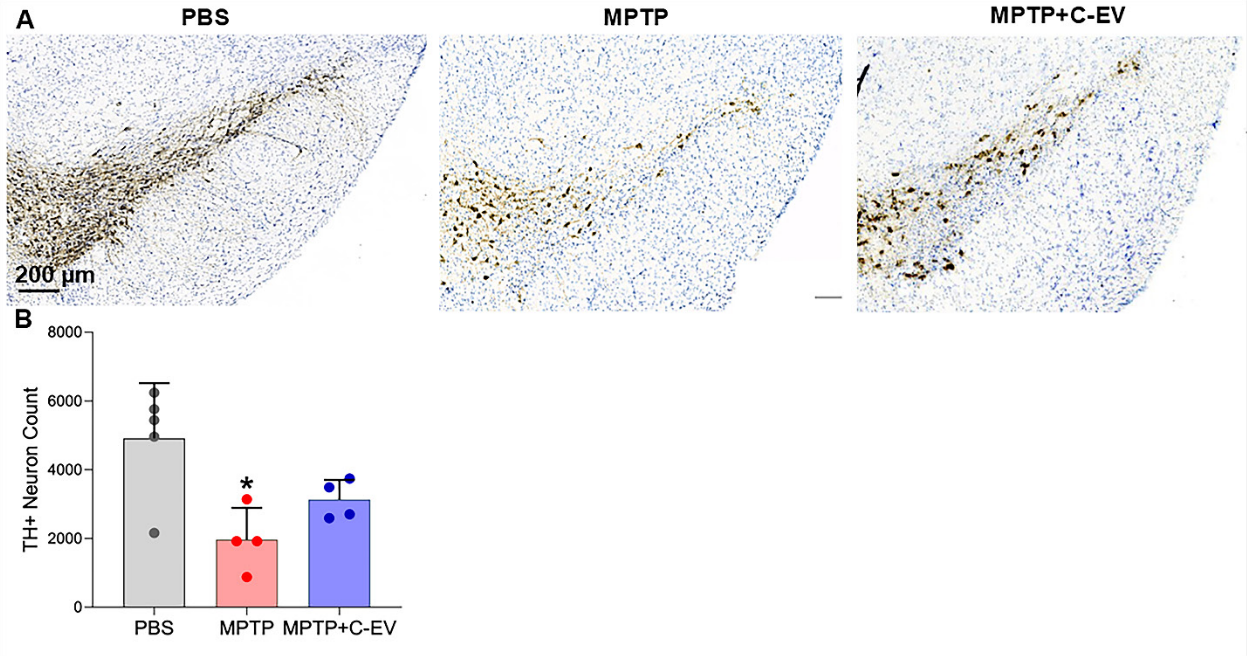

Supplementary Figure 1. First analysis of dopaminergic neurons in MPTP mice. In our initial study, mice were injected with 10^{11} EVs intravenously. EV doses were administered one and three days before and on the day of MPTP intoxication, followed by neuronal counts on day 7. (A) Representative IHC images of dopaminergic neurons (Nissl + TH+) in the substantia nigra. (B) TH+ neuron count quantified using SteroInvestigator. Data are expressed as mean ± SEM. n=5 in PBS, n=4 in MPTP, n=4 in MPTP+EV. *$p \leq 0.05$, versus PBS; #$p \leq 0.05$, versus MPTP. Abbreviations: PBS: Phosphate-Buffered Saline; MPTP: 1-Methyl-4-phenyl-1,2,3,6-tetrahydropyridine; C-EVs: Colostrum Derived Extracellular Vesicles; IHC: immunohistochemistry.

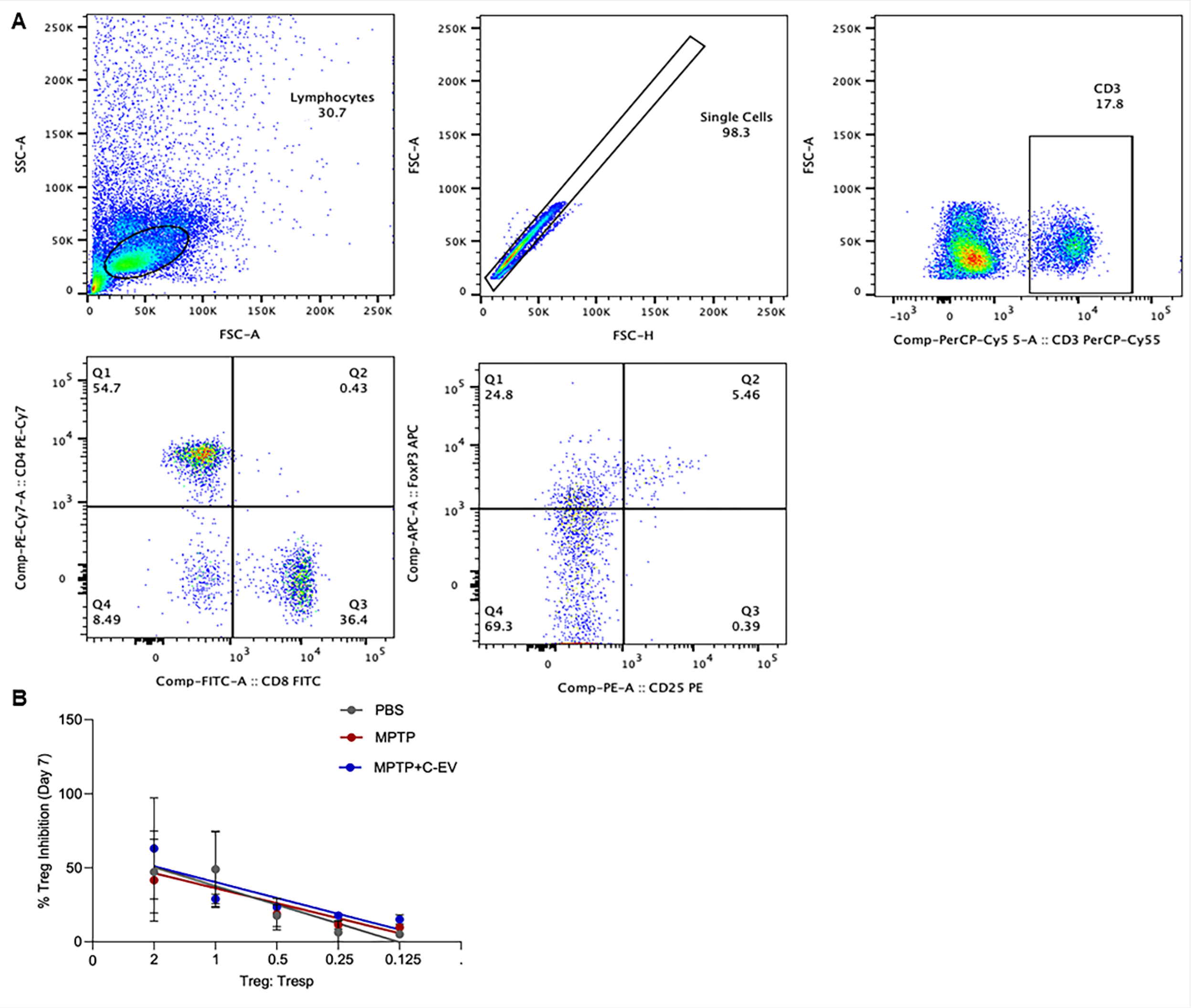

Supplementary Figure 2. Tregs in C-EV treated MPTP mice. (A) Gating strategy for Treg subpopulation analysis was performed from the parent cell population. (B) Linear regression analysis of %Treg-mediated inhibition of CFSE-stained Tresp (CD4+CD25-) cells in different treatment groups. Data are expressed as the mean ± SEM. n=5 in PBS, n=4 in MPTP, n=4 in C-EV treated MPTP mice. Abbreviations: Tresp, T responder cells; Treg: T Regulatory cells; PBS: Phosphate-Buffered Saline; MPTP: 1-Methyl-4-phenyl-1,2,3,6-tetrahydropyridine; C-EVs: Colostrum-derived extracellular vesicles.

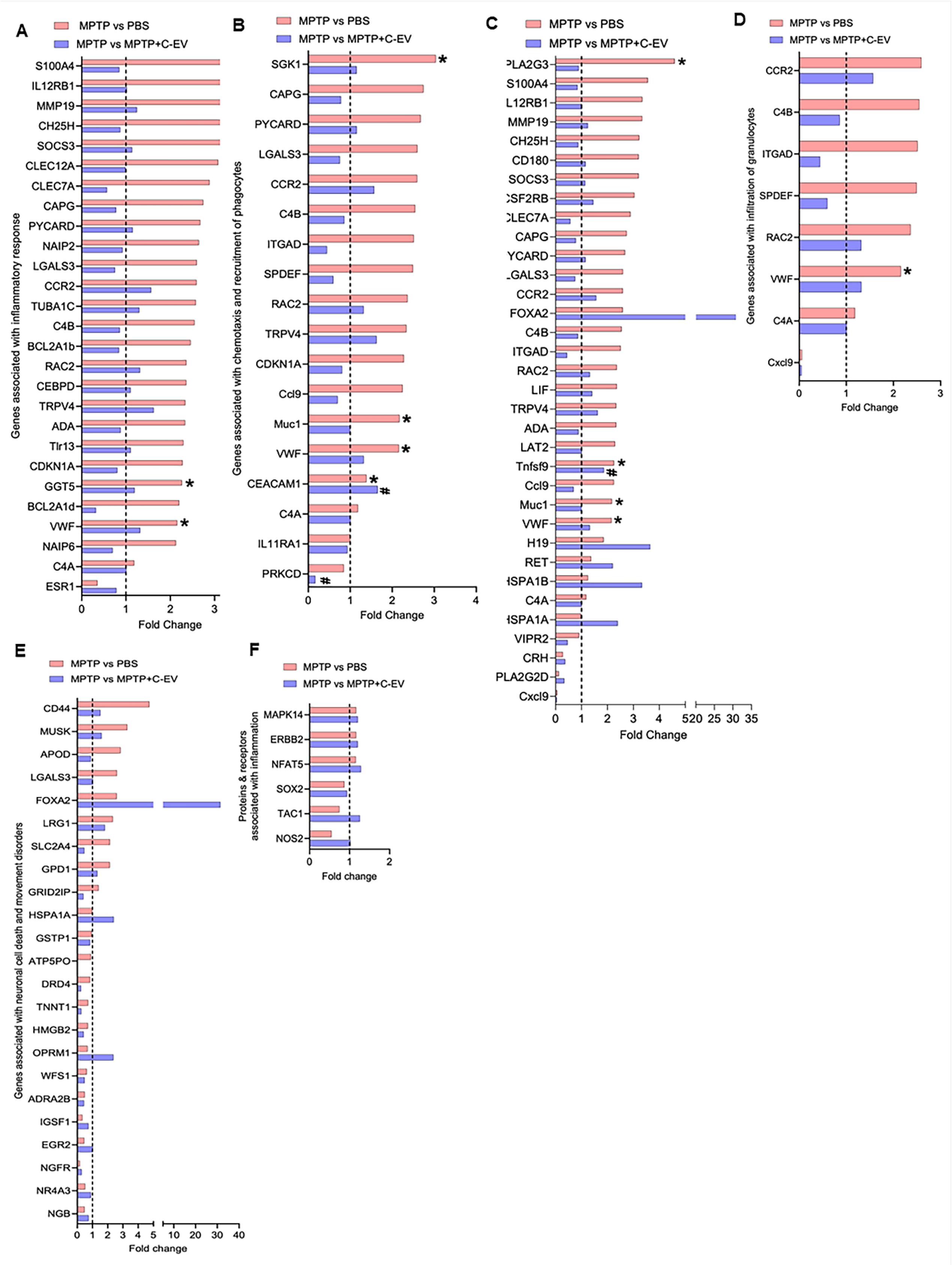
A
MPTP vs PBS
MPTP vs MPTP+C-EV
Genes associated with inflammatory response
S100A4
IL12RB1
MMP19
CH25H
SOCS3
CLEC12A
CLEC7A
CAPG
PYCARD
NAIP2
LGALS3
CCR2
TUBA1C
C4B
BCL2A1b
RAC2
CEBPD
TRPV4
ADA
Tlr13
CDKN1A
GGT5
BCL2A1d
VWF
NAIP6
C4A
ESR1
0 1 2 3
Fold Change
B
MPTP vs PBS
MPTP vs MPTP+C-EV
Genes associated with chemotaxis and recruitment of phagocytes
SGK1
CAPG
PYCARD
LGALS3
CCR2
C4B
ITGAD
SPDEF
RAC2
TRPV4
CDKN1A
Ccl9
Muc1
VWF
CEACAM1
C4A
IL11RA1
PRKCD
0 1 2 3 4
Fold Change
C
MPTP vs PBS
MPTP vs MPTP+C-EV
LA2G3
S100A4
L12RB1
MMP19
CH25H
CD180
SOCS3
SF2RB
LEC7A
CAPG
YCARD
GALS3
CCR2
FOXA2
C4B
ITGAD
RAC2
LIF
TRPV4
ADA
LAT2
Tnfsf9
Ccl9
Muc1
VWF
H19
RET
SPA1B
C4A
SPA1A
VIPR2
CRH
PLA2G2D
Cxcl9
0 1 2 3 4 5 20 25 30 35
Fold Change
D
MPTP vs PBS
MPTP vs MPTP+C-EV
Genes associated with infiltration of granulocytes
CCR2
C4B
ITGAD
SPDEF
RAC2
VWF
C4A
Cxcl9
0 1 2 3
Fold Change
E
MPTP vs PBS
MPTP vs MPTP+C-EV
Genes associated with neuronal cell death and movement disorders
CD44
MUSK
APOD
LGALS3
FOXA2
LRG1
SLC2A4
GPD1
GRID2IP
HSPA1A
GSTP1
ATP5PO
DRD4
TNNT1
HMGB2
OPRM1
WFS1
ADRA2B
IGSF1
EGR2
NGFR
NR4A3
NGB
0 1 2 3 4 5 10 20 30 40
Fold change
F
MPTP vs PBS
MPTP vs MPTP+C-EV
Proteins & receptors associated with inflammation
MAPK14
ERBB2
NFAT5
SOX2
TAC1
NOS2
0 1 2
Fold change

Supplementary Figure 3. Composite of differentially expressed genes. (A) Differential gene expression associated with inflammatory response. (B) Differential gene expression is associated with chemotaxis and the recruitment of phagocytes. (C) Differential gene expression was associated with leukocyte activation and chemotaxis. (D) Differential gene expression associated with granulocyte infiltration. (E) Differential gene expression was associated with neuronal cell death and movement disorders. (F) Differential gene expression of the proteins and receptors associated with inflammation. Data are expressed as fold changes. The dotted line indicates the PBS group. n=3 mice per group. $^{*}p \leq 0.05$, versus PBS; $^{\#}p \leq 0.05$, versus MPTP. Abbreviations: *ADA*: Adenosine Deaminase; *ADRA2B*: Adrenoceptor Alpha 2B; *APOD*: Apolipoprotein D; *ATP5PO*: ATP Synthase Peripheral Stalk Subunit OSCP; *BCL2A1b/d*: BCL2 Related Protein A1 Beta/Delta; *C4A/B*: Complement C4-A/B; *CAPG*: Capping Actin Protein, Gelsolin-Like; *Ccl9*: Chemokine Ligand 9; *CCR2*: C-C Motif Chemokine Receptor 2; *CD180*: Cluster of Differentiation 180; *CD44*: Cluster of Differentiation 44; *CDKN1A*: Cyclin Dependent Kinase Inhibitor 1A; *CEACAM1*: Carcinoembryonic Antigen-Related Cell Adhesion Molecule 1; *CEBPD*: CCAAT/Enhancer Binding Protein Delta; *CH25H*: Cholesterol 25-Hydroxylase; *CLEC12A*: C-Type Lectin Domain Family 12A; *CLEC7A*: C-Type Lectin Domain Family 7A; *CRH*: Corticotropin-Releasing Hormone; *CSF2RB*: Colony Stimulating Factor 2 Receptor Beta; *Cxcl9*: Chemokine Ligand 9; *DRD4*: Dopamine Receptor D4; *EGR2*: Early Growth Response Protein 2; *ERBB2*: Erb-B2 Receptor Tyrosine Kinase 2; *ESR1*: Estrogen Receptor 1; *FOXA2*: Forkhead Box A2; *GGT5*: Gamma-Glutamyltransferase 5; *GPD1*: Glycerol-3-Phosphate Dehydrogenase 1; *GRID2IP*: GRID2 Interacting Protein; *GSTP1*: Glutathione S-Transferase Pi 1; *H19*: H19 Imprinted Maternally Expressed Transcript; *HMGB2*: High-Mobility Group Protein B2; *HSPA1A/B*: Heat Shock Protein Family A Member 1A/B; *IGSF1*: Immunoglobulin Superfamily Member 1; *IL11RA1*: Interleukin 11 Receptor Subunit Alpha 1; *IL12RB1*: Interleukin 12 Receptor Subunit Beta 1; *ITGAD*: Integrin Subunit Alpha D; *LAT2*: Linker for Activation of T Cells Family Member 2; *LGALS3*: Galectin-3; *LIF*: Leukemia Inhibitory Factor; *LRG1*: Leucine Rich Alpha-2-Glycoprotein 1; *MAPK14*: Mitogen-Activated Protein Kinase 14; *MMP19*: Matrix Metallopeptidase 19; *Muc1*: Mucin 1, Cell Surface Associated; *MUSK*: Muscle Associated Receptor Tyrosine Kinase; *NAIP2*: NLR Family Apoptosis Inhibitory Protein 2; *NAIP6*: NLR Family Apoptosis Inhibitory Protein 6; *NGB*: Neuroglobin; *NGFR*: Nerve Growth Factor Receptor; *NOS2*: Nitric Oxide Synthase 2; *NR4A3*: Nuclear Receptor Subfamily 4 Group A Member 3; *OPRM1*: Opioid Receptor Mu 1; *PLA2G2D*: Phospholipase A2 Group IID; *PLA2G3*: Phospholipase A2 Group III; *PRKCD*: Protein Kinase C Delta; *PYCARD*: PYD And CARD Domain Containing; *RAC2*: Ras-Related C3 Botulinum Toxin Substrate 2; *RET*: Rearranged During Transfection Proto-Oncogene; *S100A4*: S100 Calcium Binding Protein A4; *SGK1*: Serum/Glucocorticoid Regulated Kinase 1; *SLC2A4*: Solute Carrier Family 2 Member 4; *SOCS3*: Suppressor of Cytokine Signaling 3; *SOX2*: SRY-Box Transcription Factor 2; *SPDEF*: SAM Pointed Domain Containing ETS Transcription Factor; *TAC1*: Tachykinin Precursor 1; *Tlr13*: Toll-Like Receptor 13; *Tnfsf9*: Tumor Necrosis Factor Superfamily Member 9; *TNNT1*: Troponin T1, Slow Skeletal Type; *TRPV4*: Transient Receptor Potential Cation Channel Subfamily V Member 4; *TUBA1C*: Tubulin Alpha 1C; *VIPR2*: Vasoactive Intestinal Peptide Receptor 2; *VWF*: Von Willebrand Factor; *WFS1*: Wolfram Syndrome 1.
